# Supplementary material for: Structure-guided disulfide engineering restricts antibody conformation to elicit TNFR agonism
Source: Nat Commun. 2025 Apr 12;16:3495. doi: 10.1038/s41467-025-58773-8 (PMC11993666; doi:10.1038/s41467-025-58773-8)
Supplement: Supplementary file 1 — Supplementary Information [file 41467_2025_58773_MOESM1_ESM.pdf]

Supplementary Information to

Structure-guided disulfide engineering restricts antibody conformation to elicit TNFR agonism

Isabel G. Elliott[1,2,3,4,#], Hayden Fisher[1,2,3,4,5,#], H.T. Claude Chan[3], Tatyana Inzhelevskaya[3], C. Ian Mockridge[3], Christine A. Penfold[3], Patrick J. Duriez[3], Christian M. Orr[6], Julie Herniman[1], Kri T. J. Müller[3], Jonathan W. Essex[1,4,‡], Mark S. Cragg[3,4,‡], Ivo Tews[2,4,‡,*]

1 University of Southampton, School of Chemistry; Southampton SO17 1BJ, UK.
2 University of Southampton, Biological Sciences; Southampton SO17 1BJ, UK.
3 University of Southampton, Centre for Cancer Immunology; Southampton, SO16 6YD, UK.
4 University of Southampton, Institute for Life Sciences; Southampton SO17 1BJ, UK.
5 European Synchrotron Radiation Facility, Grenoble, Cedex 9, 38043, France
6 Diamond Light Source, Didcot, OX11 0FA, UK.

* Corresponding author. Email: Ivo.Tews@soton.ac.uk
# These authors contributed equally
‡ These authors jointly supervised this work

This supplement contains:
Supplementary Methods
Supplementary Tables 1-15
Supplementary Figures 1-23
Supplementary References

Supplementary Methods

Surface plasmon resonance (SPR)

Binding kinetics of mAb were analysed by SPR using the Biacore T200 instrument. Recombinant soluble *h*CD40-hFc-His or *h*4-1BB-*h*Fc-His (both from R&D Systems Inc.) was immobilised onto a CM5 sensor chip via amine coupling at a target level of 500 RU, according to the manufacturer's protocol. Anti-*h*CD40 or anti-*h*4-1BB mAb were injected through the flow cells at 100, 20, 4, 0.8, 0.16 and 0 nM in HBS-EP+ running buffer (0.01M HEPES, 0.15M NaCl, 3 mM EDTA, 0.005% Surfactant P20, pH 7.4) at a flow rate of 30 μL/min at 25 °C, with 300 seconds for association and 300 s for dissociation. Regeneration of the chip was performed for 30 s at a flow rate of 30 μL/min using 10 mM glycine, pH 1.5. Sensorgrams were fitted with the bivalent binding model and k_a and k_d were calculated using the Biacore Bioevaluation software. K_D was calculated as k_d/k_a. All reagents, equipment and software were obtained from Cytiva, unless otherwise specified.

SDS-PAGE

mAb and F(ab')$_2$ fragments were analysed by SDS-PAGE. Reduced samples were prepared using 5 μg protein with NuPAGE LDS Sample buffer (4x) and NuPAGE Sample Reducing agent (10x) (Invitrogen, ThermoFisher UK). Non-reduced samples were prepared as above but without reducing agent. Samples were heated at 70 °C for 10 minutes prior to running with a Novex Sharp pre-stained protein standard ladder (Invitrogen, ThermoFisher UK) on a pre-cast 4-12% NuPAGE Bis-Tris protein gel (Invitrogen, ThermoFisher UK) with MOPS running buffer (MOPS, TRIS, SDS, EDTA, pH 7.7) at 150 V. Gels were stained with Coomassie blue stain (0.1% Coomassie Blue R-250 (Sigma), 50% MeOH, 10% acetic acid) and de-stained with 10% acetic acid (VWR Chemicals).

CE-SDS

Capillary electrophoresis with sodium dodecyl sulfate (CE-SDS) was used to characterise mAb and F(ab')$_2$ fragments. Reduced and non-reduced samples were prepared using the IgG Purity and Heterogeneity Assay Kit, according to manufacturer's instructions, and samples were run on a PA 800 Plus Pharmaceutical Analysis System. All equipment and reagents from SCIEX.

Mass spectrometry

F(ab')$_2$ fragments were characterised by mass spectrometry to determine the pepsin cleavage site in the hinge region, to allow for the correct sequence to be modelled by MD simulations. Samples (n=1) were analysed using a Bruker MaXis (Bruker Daltonics, Bremen, Germany) time of flight (TOF) mass spectrometer. 5 uL of sample was injected into a Dionex Ultimate 3000 autosampler coupled to an ultrahigh performance liquid chromatography (uHPLC) pump and variable wavelength detector. uHPLC was achieved using a Waters UPLC BEH C18 (50 mm x 2.1 mm x 1.7 μm) column heated to 50°C (Waters, Wilmslow, UK). Gradient elution from 5% acetonitrile (0.2% formic acid) to 100% acetonitrile (0.2 % formic acid) was performed in five minutes at a flow rate of 0.6 mL/min. High resolution positive electrospray ionisation mass spectra (m/z 500 to 3000) were recorded using OtofControl Acquisition Program version 3.2.41.0 and Instrument Program version 5.3.14.0. The system was calibrated using sodium formate in enhanced quadratic mode. Source conditions were as follows, endplate offset -500 V, capillary 4500 V, nebuliser gas 4.0 Bar, drying gas 6.0 L/min, drying temperature 230°C. Multiply charged data were processed using Max Ent Deconvolution using DataAnalysis version 4.0.275.0. The cleavage site was determined by calculating the predicted masses of F(ab')$_2$ fragments to most closely match the mass spectrometry derived F(ab')$_2$ mass (Supplementary Figure 21).

Nano Differential Scanning Fluorimetry

IgG were characterised by nano differential scanning fluorimetry (NanoDSF) using the Prometheus NT.48 (NanoTemper Technologies, Munich, Germany). Samples were loaded into Prometheus standard capillaries and exposed to thermal stress from 15°C to 95°C, at a ramp of 1°C/min. Intrinsic protein

fluorescence was collected at 330 nm and 350 nm. Thermal stability parameters (unfolding temperatures, T_{onset} and T_m) were calculated by PR. ThermControl software (NanoTemper Technologies) using first derivative analysis of 350 nm/330 nm fluorescence ratio was plotted against temperature.

Crystallographic data collection, processing, structure determination and refinement

Crystals were cryo-protected in mother liquor containing 20% glycerol and harvested using litholoops (Molecular Dimensions), then cryo-cooled in liquid nitrogen. Standard X-ray diffraction data for the cross-over + T222C κE123C crystals were collected at 100 K at beamline ID30A-3 at the European Synchrotron Radiation Facility (ESRF, Grenoble, France) operating at 0.9677 Å, using an Eiger X 4M detector[1]. 1200 images were collected using a rotation increment of 0.1° with a 0.05 s exposure per image. Anomalous diffraction data for the cross-over + T222C κE123C and the cross-over + K228C crystals were collected at 50 K at the in-vacuo long wavelength beamline I23 at the Diamond Light Source (DLS, Oxford, UK) operating at 2.76 Å to maximise the anomalous sulfur signal, using a Pilatus 12M detector[2]. 3600 images were collected using a rotation increment of 0.1° with 0.1 s exposure per image. Data were integrated using either GrenADES parallelproc[3] which performs integration using XDS[4] and scaling and merging with XSCALE[4] and POINTLESS[5], or XIA2 DIALS[6]. Data manipulation, molecular replacement and refinement were carried out within the CCP4i2 graphical user interface of the CCP4 program suite[7,8]. Molecular replacement was performed using MOLREP[9] with the PDB model 6TKE as the homologous search model. Iterative model building and refinement were carried out using Coot[10] and REFMAC5[11], respectively. The cross-over + K228C data was refined using global non-crystallographic symmetry restraints. Explicit translation-libration-screw (TLS) coefficients were used in refinement of both structures. The PDB-REDO server[12] was used to generate restraints for use in refinement. MolProbity[13] and the PDB validation server[14] were used for model validation prior to deposition. The final models were deposited in the PDB[14] with accession codes 8PUK (cross-over + T222C κE123C) and 8PUL (cross-over + K228C). The sulfur anomalous maps were obtained using either phenix.refine[15] or the anomalous and heavy-atom density calculation software (ANODE)[16] which uses fully refined structures to calculate anomalous difference Fourier maps. PyMOL was used to prepare figures depicting protein structure.

SAXS structure ensemble fitting

The program GAJOE (genetic algorithm judging optimisation of ensembles)[17] was used to select ensembles of conformations from the MD-generated starting pool of structures. FFMAKER (form-factor maker) was first run to compute the scattering amplitudes of the structures in the starting pool. GAJOE was then run with the minimum selected ensemble size set to 1 and the maximum selected ensemble size set to 50, with the remainder of the settings set to default: 100 genetic algorithm cycles were performed per run, each cycle comprised 1000 generations, the number of ensembles per generation was 50, curve repetition was enabled. The program was run 10 independent times to ensure convergence of the genetic algorithm. The population density curves were plotted using Python, and representative states were visualised using PyMOL. Error-weighted residuals (Δ/σ) were calculated using I(exp)-I(model)/ σ.

Hinge and torsion angles

Hinge and torsion angles of F(ab')$_2$ fragments were calculated in PyMOL, by defining multiple centres of mass (COM), based on all atoms, and calculating the angles between them (Supplementary Figure 9). The torsion angle was calculated using the dihedral measurement tool in PyMOL as the angle between the COM of the variable region in one F(ab') arm to the COM of the constant domain of that F(ab') arm to the COM of the constant domain of the second F(ab') arm to the variable region of the second F(ab'). The hinge angle was calculated using the angle measurement tool in PyMOL as the angle between the COM of the variable region of one F(ab') arm to the COM of the constant domain including the hinge for both F(ab') arms to the COM of the variable region of the second F(ab') arm.

The variable region contained heavy chain amino acids 1-122, light chain amino acids 1-107 and the constant domain contained heavy chain amino acids 123-220, light chain amino acids 108-214. The constant domain including the hinge for the hinge angle calculation contained heavy chain amino acids 123-231, light chain amino acids 108-214.

Supplementary Table 1: Antibody production details.

ID number	Antibody variant	SEC	Final Yield (mg)	Transfection Volume (mL)	Titre (mg/mL)	Aggregation (%) [1]	Production method
Batch 2	ChiLob7/4 *h*IgG2 cross-over +K228C	N	16.5	50	0.33	0	Transient
Batch 2	ChiLob7/4 *h*IgG2 cross-over +T222C κE123C	N	10.6	50	0.21	0.9	Transient
Batch 1	SAP1.3 *h*IgG2 C232S+C233S (opt)	N	102.3	200	0.51	0.7	Transient
Batch 1	SAP1.3 *h*IgG2 C232S κC214S (opt)	N	104.1	100	1.0	0.5	Transient
Batch 1	SAP1.3 *h*IgG2 C233S κC214S (opt)	N	47.6	100	0.48	0.6	Transient
Batch 1	SAP1.3 *h*IgG2 (opt)	N	16.1	50	0.32	0	Transient
Batch 2	SAP1.3 *h*IgG2 C232S κC214S	N	10.4	200	0.05	0	Transient
Batch 2	SAP1.3 *h*IgG2 C232S+C233S	N	13.0	200	0.07	0	Transient
Batch 1	ChiLob7/4 *h*IgG2 cross-over +T222C κE123C	N	16.3	100	0.16	0.7	Transient
Batch 1	ChiLob7/4 *h*IgG2 cross-over +K228C	N	16.1	50	0.32	0	Transient
Batch 1	SAP1.3 *h*IgG2 C233S κC214S	N	2.9	50	0.06	0	Transient
Batch 1	SAP1.3 *h*IgG2 C232S κC214S	N	2.56	50	0.05	0	Transient
Batch 1	SAP1.3 *h*IgG2 C232S+C233S	N	3.9	50	0.08	0	Transient
Batch 1	SAP1.3 *h*IgG2	N	15	500	0.03	1.1	Transient – shaker produced in HEK293F cells
Batch 1	ChiLob7/4 *h*IgG2	Y	215	2100	0.1	0.4	Produced in Shaker Flask - CHO-K1 cells adapted to serum-free media
Batch 1	ChiLob7/4 *h*IgG2 C232S κC214S	Y	158	2100	0.08	0.4	Produced in Shaker Flask - CHO-K1 cells adapted to serum-free media
Batch 1	ChiLob7/4 *h*IgG2 C233S κC214S	Y	169	1900	0.09	1.0	Produced in Shaker Flask - CHO-K1 cells adapted to serum-free media
Batch 1	ChiLob7/4 *h*IgG2 C232S+C233S	Y	45.5	5800	0.008	0.1	Produced in Large Flask static - CHO-K1 GMEMS +dFCS Bulk Culture
Batch 1	ChiLob7/4 *h*IgG1	Y	235	1960	0.11	1.5	Produced in Shaker Flask - CHO-K1 cells adapted to serum-free media
Batch 1	SAP1.3 *h*IgG1	N	77.1	1850	0.04	0.5	Produced in Shaker Flask - CHO-K1 cells adapted to serum-free media

[1] Aggregation was calculated as subtraction of the aggregation peak area from the elution peak area. Corresponding HPLC traces are shown in Supplementary Figure 1.

Supplementary Table 2: Melting points for anti-*h*CD40 ChiLob7/4 *h*IgG2 variants as IgG.
NanoDSF was used to measure thermal stability of *h*IgG2 variants. Table shows the melting temperature calculated from three repeats, with an average of the three repeats. Melting curves are shown in Supplementary Figure 3.

ChiLob7/4 antibody variant	Melting temperature, T_m (°C)		
	Run 1	Run 2	Run 3
*h*IgG2	67.8	67.8	67.8
C232S+C233S	68.7	68.7	68.8
C232S κC214S (cross-over)	66.7	66.7	66.7
cross-over + T222C κE123C	68.4	68.4	68.4
cross-over + K228C	69.1	69.1	69.2

Supplementary Table 3: Binding affinity of anti-*h*CD40 ChiLob7/4 and anti-*h*4-1BB SAP1.3 *h*IgG2 C-S variants.

Surface plasmon resonance (SPR) was used to measure binding kinetics of variants. Recombinant soluble *h*CD40-*h*Fc-His or *h*4-1BB-*h*Fc-His was immobilised on a CM5 sensor chip by amine coupling at 500 RU, and variants as IgG were injected over at a range of different concentrations (100, 20, 4, 0.8, 0.16 nM) at 25°C. **a** Table shows for ChiLob7/4 and SAP1.3, the k_a(association/on rate), k_d (dissociation/off rate) and K_D (k_d/k_a, Equilibrium dissociation constant). Values calculated from fitting sensorgrams to bivalent binding model and show mean ± range from 2 independent experiments. **b** Table shows Rmax and χ^2 values from the 2 experiments. Exemplar raw data shown in Supplementary Figure 4.

a

	**ChiLob7/4**			**SAP1.3**		
	k_a ($\times10^4$ $M^{-1}s^{-1}$)	k_d ($\times10^{-4}$ s^{-1})	K_D ($\times10^{-9}$ M)	k_a ($\times10^4$ $M^{-1}s^{-1}$)	k_d ($\times10^{-4}$ s^{-1})	K_D ($\times10^{-9}$ M)
*h*IgG1	13.0 ± 0.70	0.747 ± 0.165	0.579 ± 0.159	4.96 ± 0.95	3.72 ± 0.14	6.65 ± 0.99
C232S+C233S	11.3 ± 2.20	0.705 ± 0.415	0.611 ± 0.248	5.86 ± 0.61	2.89 ± 0.19	4.93 ± 0.19
C233S κC214S	5.63 ± 1.62	3.72 ± 0.620	6.67 ± 0.817	6.08 ± 0.29	6.22 ± 0.26	10.24 ± 0.06
C232S κC214S	6.18 ± 0.99	2.94 ± 0.670	4.74 ± 0.325	6.12 ± 0.15	8.20 ± 0.30	13.41 ± 0.16
*h*IgG2	8.33 ± 0.94	1.64 ± 0.0600	1.97 ± 0.151	5.61 ± 0.61	7.72 ± 0.07	13.81 ± 1.63

b

	**ChiLob7/4**				**SAP1.3**			
	Rmax exp1 (RU)	Rmax exp2 (RU)	χ^2 exp1	χ^2 exp2	Rmax exp1 (RU)	Rmax exp2 (RU)	χ^2 exp1	χ^2 exp2
*h*IgG1	629.8	633	4.99	2.44	543.3	549.9	4.52	1.79
C232S+C233S	635.8	648.4	35.8	14	557.6	569.7	5.51	1.08
C233S κC214S	586.6	585.9	27.4	17.1	517.4	517.5	5.17	5.4
C232S κC214S	605.6	613.5	25.8	19.9	529.8	530.4	5.31	3.81
*h*IgG2	607.4	647.3	23.1	20.9	509.2	526.4	7.61	2.72

Supplementary Table 4: Data from SAXS analysis of anti-*h*CD40 ChiLob7/4 F(ab')$_2$ variants.

Antibody	ChiLob7/4						
				cross-over		cross-over + K228C	cross-over + T222C κE123C
Kabat	*h*IgG1	C232S+C233S	C233S κC214S	C232S κC214S	*h*IgG2	C232S+K228C κC214S	C232S+T222C κE123C+κC214S
(a) SAS-derived structural parameters							
Methods/Software	BioXTAS RAW 2.3.0[18] for Guinier analysis, GNOM from ATSAS 4.0.1-1[19] implemented in BioXTAS RAW 2.3.0 for P(r) analysis						
Guinier analysis							
I(0) (cm^{-1})	97.01 ± 0.24	90.34 ± 0.17	88.99 ± 0.19	83.69 ± 0.17	46.81 ± 0.19	103.49 ± 0.21	84.45 ± 0.16
R_g (Å)	45.59 ± 0.16	42.11 ± 0.11	40.06 ± 0.12	38.90 ± 0.12	39.13 ± 0.23	38.89 ± 0.13	38.49 ± 0.11
qR_g range	0.5733-1.3052	0.3114- 1.2929	0.4-1.2921	0.3280-1.2950	0.3299-1.3027	0.4688-1.2945	0.2647-1.3012
(datapoint range)	16-47	6-51	11-54	8-56	8-56	15-56	5-57
Linear fit assessment (R^2)	0.9976	0.9976	0.9959	0.9976	0.9910	0.9963	0.9973
P(r) analysis							
I(0) (cm^{-1})	98.99 ± 0.23	90.98 ± 0.16	89.39 ± 0.17	84.28 ± 0.18	47.20 ± 0.15	103.50 ± 0.21	84.76 ± 0.14
R_g (Å)	48.55 ± 0.15	43.56 ± 0.10	41.12 ± 0.10	40.0 ± 0.12	40.37 ± 0.20	39.45 ± 0.11	39.22 ± 0.10
D_{max} (Å)	174	148	141	139	137	132	132
q range ($Å^{-1}$)	0.0126-0.3	0.0074-0.3	0.01-0.3	0.0084-0.3	0.0084-0.3	0.0121-0.3	0.0069-0.3
P(r) fit assessment (Quality estimate, χ^2)	0.7385, 1.0893	0.8072, 1.0524	0.825, 1.0652	0.8536, 1.0530	0.8462, 1.0006	0.7980, 1.2127	0.8123, 1.0525

Antibody	ChiLob7/4						
				cross-over		cross-over + K228C	cross-over + T222C κE123C
Kabat	*h*IgG1	**C232S+C233S**	**C233S κC214S**	**C232S κC214S**	***h*IgG2**	**C232S+K228C κC214S**	**C232S+T222C κE123C+κC214S**
(b) Scattering particle size							
Methods/Software	BioXTAS RAW 2.3.0[18] for Porod volume and concentration-independent MW methods (Vc[20], MoW[21], Shape&Size[22], Bayesian inference[23]) Protparam (https://web.expasy.org/protparam/)[24] for MW from chemical composition						
Volume estimates (Å^3)							
Porod volume (ratio to M)	133000 (1.35)	127000 (1.29)	125000 (1.27)	126000 (1.28)	123000 (1.25)	124000 (1.26)	124000 (1.26)
Molecular weight (MW) estimates (Da)							
From chemical composition	98250	98696	98696	98696	98760	98656	98648
From concentration-independent method (kDa)							
Bayesian inference,	94.2	91.2	85.7	94.2	85.7	94.2	94.2
% confidence	68.3	42.1	32.2	88	36.1	82.3	45.1
(range, % confidence)	89.7-99.2, 96.1	84.2-95.8, 97.5	84.3-95.8, 93.5	89.7-95.8, 95.1	77.4-92.7, 93.8	89.7-95.8, 97.2	87-95.8, 93.8
MoW	110.1	105.1	103.6	104.3	101.9	103.3	102.9
Vc	84.2	83.7	83.3	86.8	83.1	85.5	85.9
Size and shape	nd	99.6	105.7	94.6	96.6	93.3	95

Antibody	ChiLob7/4						
				cross-over		cross-over + K228C	cross-over + T222C κE123C
Kabat	*h*IgG1	C232S+C233S	C233S κC214S	C232S κC214S	***h*IgG2**	C232S+K228C κC214S	C232S+T222C κE123C+κC214S
(c) Modelling (to the crystal structure)							
Software	CRYSOL from ATSAS 3.2.1[19] with constant subtraction enabled, 50 spherical harmonics, explicit hydrogens and a water shell						
Crystal structure PDB entry[a]	N/A	N/A	6TKF	6TKE	N/A	8PUL	8PUK
q range for fit	N/A	N/A	0.01-0.2	0.0084-0.2	N/A	0.0121-0.2	0.0069-0.2
χ^2, CorMap p-value	N/A	N/A	9.289, 1.02e-18	8.559, 5.15e-19	N/A	2.901, 1.56e-07	7.398, 5.2e-19
Predicted R_g (Å)	N/A	N/A	36.95	36.65	N/A	37.82	36.59
Envelope diameter (Å)	N/A	N/A	125.2	124.5	N/A	129.5	123.6
(d) Modelling (to the best fitting single models from the molecular dynamics simulations)							
Software	CRYSOL from ATSAS 3.2.1[19] with constant subtraction enabled, 50 spherical harmonics, explicit hydrogens and a water shell						
q range for fit	N/A	0.0074-0.2	0.01-0.2	0.0084-0.2	N/A	0.0121-0.2	0.0069-0.2
χ^2, CorMap p-value	N/A	1.607, 2.09e-05	1.232, 0.0215	1.081, 0.515	N/A	1.311, 0.16	1.009, 0.518
Predicted R_g (Å)	N/A	42.34	40.45	39.57	N/A	38.93	39.07
Envelope diameter (Å)	N/A	142.1	138.1	137.5	N/A	133.6	136.4
Hinge angle (°)	N/A	135.884	132.456	126.648	N/A	125.314	124.885
Torsion angle (°)	N/A	86.66	69.406	60.339	N/A	62.83	62.088

Antibody	ChiLob7/4						
				cross-over		cross-over + K228C	cross-over + T222C κE123C
Kabat	*h*IgG1	C232S+C233S	C233S κC214S	C232S κC214S	*h*IgG2	C232S+K228C κC214S	C232S+T222C κE123C+κC214S
(d) Ensemble optimisation							
Software	EOM GAJOE v2.1 (with default parameters, constant subtraction enabled, curve repetition allowed, min 1 curve, max 50 curves, 6000 models in MD-generated pool)						
q range for fit	N/A	0.0074-0.2	0.01-0.2	0.0084-0.2	N/A	0.0121-0.2	0.0069-0.2
χ^2, CorMap p-value	N/A	1.028, 0.0433	1.193, 0.0215	1.087, 0.301	N/A	1.306, 0.16	1.012, 0.0219
No of curves	N/A	6001	6001	6001	N/A	6001	6001
R_{flex} (%) (random)	N/A	79.59 (**76.32**)	61.71 (**78.57**)	52.25 (67.97)	N/A	47.05 (65.67)	35.46 (74.82)
R_σ	N/A	1.71	1.48	1.1	N/A	0.96	0.3
Ensemble R_g (Å) ± SD	N/A	42.87 ± 3.27	40.26 ± 0.97	39.16 ± 0.49	N/A	38.85 ± 0.28	38.8 ± 0.35
Ensemble D_{max} (Å) ± SD	N/A	141.43 ± 13.56	138.81 ± 6.05	133.92 ± 3.16	N/A	131.95 ± 0.46	132.42 ± 2.98
Hinge angle ° ± SD	N/A	135.61 ± 20.24	128.06 ± 4.1	126.45 ± 4.88	N/A	126.11 ± 0.56	126 ± 4.19
Variance	N/A	380.56	15.3	21.87	N/A	0.27	14.63
Torsion angle ° ± SD	N/A	105.35 ± 48.33	50.82 ± 9.32	58.62 ± 4.19	N/A	67.32 ± 5.34	65.32 ± 2.72
Variance	N/A	2169.17	79.03	16.07	N/A	24.97	6.16

Supplementary Table 5: Data from SAXS analysis of anti-*h*4-1BB SAP1.3 $F(ab')_2$ variants.

Antibody	**SAP1.3**				
Kabat	***h*IgG1**	**C232S+C233S**	**C233S κC214S**	**C232S κC214S**	***h*IgG2**
(a) SAS-derived structural parameters					
Methods/Software	BioXTAS RAW 2.3.0[18] for Guinier analysis, GNOM from ATSAS 4.0.1-1[19] implemented in BioXTAS RAW 2.3.0 for P(r) analysis				
Guinier analysis					
I(0) (cm^{-1})	51.78 ± 0.06	34.18 ± 0.05	57.69 ± 0.04	46.42 ± 0.06	53.43 ± 0.04
R_g (Å)	44.95 ± 0.08	41.64 ± 0.08	38.90 ± 0.05	38.31 ± 0.08	38.30 ± 0.04
qR_g range	0.2626-1.2870	0.2864-1.3000	0.2474-1.2949	0.4421-1.2953	0.1642-1.2950
(datapoint range)	3-47	5-52	4-56	14-57	0-57
Linear fit assessment (R^2)	0.9991	0.9981	0.9995	0.9993	0.9987
P(r) analysis					
I(0) (cm^{-1})	52.50 ± 0.7	34.57 ± 0.04	57.93 ± 0.04	46.69 ± 0.06	53.65 ± 0.04
R_g (Å)	47.20 ± 0.08	43.36 ± 0.09	39.66 ± 0.05	39.12 ± 0.06	38.99 ± 0.03
D_{max} (Å)	163	151	137	129	128
q range ($Å^{-1}$)	0.0058-0.3	0.0069-0.3	0.0064-0.3	0.0115-0.3	0.0043-0.3
P(r) fit assessment (Quality estimate, χ^2)	0.7899, 1.1426	0.8342, 1.0954	0.7862, 0.95	0.9077, 1.0854	0.9250, 1.1386

Antibody	SAP1.3				
Kabat	*h*IgG1	**C232S+C233S**	**C233S κC214S**	**C232S κC214S**	***h*IgG2**
(b) Scattering particle size					
Methods/Software	BioXTAS RAW 2.3.0[18] for Porod volume and concentration-independent MW methods (Vc[20], MoW[21], Shape&Size[22], Bayesian inference[23]) Protparam (https://web.expasy.org/protparam/)[24] for MW from chemical composition				
Volume estimates (Å^3)					
Porod volume (ratio to M)	139000 (1.40)	140000 (1.41)	135000 (1.36)	130000 (1.30)	126000 (1.27)
Molecular weight (MW) estimates (Da)					
From chemical composition	99000	99446	99446	99446	99510
From concentration-independent method (kDa)					
Bayesian inference,	94.2	101	94.2	94.2	85.7
% confidence	92.7	59.4	45	73.3	31.9
(range, % confidence)	89.7-95.8, 97.5	92.7-106.9, 95.6	92.7-106.9, 91.8	92.7-99.2, 91.3	81.9-92.7, 31.9
MoW	115.2	116.5	112.1	107.9	104.3
Vc	88.2	99.6	93.5	90.8	85.6
Size and shape	95.5	102.2	95.1	97.5	97.5

Supplementary Table 6: Data from SAXS analysis of anti-*h*CD40 ChiLob7/4 IgG variants.

Antibody	ChiLob7/4						
				cross-over		cross-over + K228C	cross-over + T222C κE123C
Kabat	*h*IgG1	C232S+C233S	C233S κC214S	C232S κC214S	*h*IgG2	C232S+K228C κC214S	C232S+T222C κE123C+κC214S
(a) SAS-derived structural parameters							
Methods/Software	BioXTAS RAW 2.3.0[18] for Guinier analysis, GNOM from ATSAS 4.0.1-1[19] implemented in BioXTAS RAW 2.3.0 for P(r) analysis						
Guinier analysis							
I(0) (cm^{-1})	278.81 ± 0.26	375.39 ± 0.28	255.00 ± 0.25	162.48 ± 0.24	121.20 ± 0.19	237.15 ± 0.19	187.77 ± 0.23
R_g (Å)	50.65 ± 0.07	50.31 ± 0.06	49.41 ± 0.08	48.80 ± 0.11	49.32 ± 0.12	48.71 ± 0.06	48.50 ± 0.09
qR_g range	0.3787-1.2976	0.3515-1.2889	0.3210-1.2660	0.3649-1.2981	0.3446-1.2878	0.3643-1.2958	0.2913-1.3139
(datapoint range)	3-40	2-40	1-40	3-42	2-41	3-42	0-43
Linear fit assessment (R^2)	0.9996	0.9996	0.9995	0.999	0.9987	0.9996	0.9985
P(r) analysis							
I(0) (cm^{-1})	278.6 ± 0.24	375.2 ± 0.26	254.5 ± 0.21	162.2 ± 0.22	120.6 ± 0.17	236.8 ± 0.16	187.5 ± 0.22
R_g (Å)	50.83 ± 0.07	50.57 ± 0.04	49.39 ± 0.05	48.92 ± 0.08	49.080 ± 0.09	48.80 ± 0.04	48.69 ± 0.06
D_{max} (Å)	166	165	160	160	160	153	151
q range ($Å^{-1}$)	0.0075-0.3002	0.007-0.3002	0.0065-0.3002	0.0075-0.3002	0.007-0.3002	0.0075-0.3002	0.006-0.3002
P(r) fit assessment (Quality estimate, χ^2)	0.8744, 1.1181	0.8634, 1.1316	0.8442, 1.2219	0.9413, 1.0358	0.9064, 1.1804	0.9067, 1.2231	0.9119, 1.1476

Antibody	ChiLob7/4						
				**cross-over**		**cross-over + K228C**	**cross-over + T222C κE123C**
Kabat	***h*IgG1**	**C232S+C233S**	**C233S κC214S**	**C232S κC214S**	***h*IgG2**	**C232S+K228C κC214S**	**C232S+T222C κE123C+κC214S**
(b) Scattering particle size							
Methods/Software	BioXTAS RAW 2.3.0[18] for Porod volume and concentration-independent MW methods (Vc[20], MoW[21], Shape&Size[22], Bayesian inference[23]) Protparam (https://web.expasy.org/protparam/)[24] for MW from chemical composition						
Volume estimates (Å^3)							
Porod volume (ratio to M)	214000 (1.46)	215000 (1.47)	209000 (1.43)	204000 (1.40)	202000 (1.38)	204000 (1.40)	205000 (1.41)
Molecular weight (MW) estimates (Da)							
From chemical composition	146328	145821	145821	145821	145886	145771	145774
From concentration-independent method (kDa)							
Bayesian inference,	146.8	169.6	157.1	138.2	130.9	157.1	157.1
% confidence	54.2	67.5	72.6	45.6	44.6	56.5	82.6
(range, % confidence)	142.2-162.7, 93.9	151.4-194.9, 96.6	142.2-176.6, 99.4	127.5-151.4, 97.6	116-142.2, 92.4	142.2-176.6, 96.3	142.2-162.7. 95.9
MoW	177.8	178.6	173.6	169.4	167.4	169.4	170.5
Vc	146.4	151.5	145.7	140.6	133	143.7	144.9
Size and shape	163.5	176.9	193.8	180.4	187.5	167.1	161.2

Supplementary Table 7: Data from SAXS analysis of anti-*h*4-1BB SAP1.3 IgG variants.

Antibody	SAP1.3				
Kabat	***h*IgG1**	**C232S+C233S**	**C233S κC214S**	**C232S κC214S**	***h*IgG2**
(a) SAS-derived structural parameters					
Methods/Software	BioXTAS RAW 2.3.0[18] for Guinier analysis, GNOM from ATSAS 4.0.1-1[19] implemented in BioXTAS RAW 2.3.0 for P(r) analysis				
Guinier analysis					
I(0) (cm^{-1})	258.24 ± 0.32	259.54 ± 0.21	229.84 ± 0.24	211.20 ± 0.25	192.27 ± 0.18
R_g (Å)	51.55 ± 0.17	50.11 ± 0.06	49.00 ± 0.08	49.00 ± 0.09	48.90 ± 0.07
qR_g range	0.3096-1.2954	0.3501-1.2838	0.3664-1.3034	0.3424-1.3033	0.3656-1.3007
(datapoint range)	0-39	2-40	3-42	2-42	3-42
Linear fit assessment (R^2)	0.9980	0.9993	0.9996	0.9993	0.9996
P(r) analysis					
I(0) (cm^{-1})	257.2 ± 0.27	259.0 ± 0.19	230.0 ± 0.18	211.0 ± 0.20	192.0 ± 0.16
R_g (Å)	51.42 ± 0.07	50.09 ± 0.04	49.31 ± 0.05	49.12 ± 0.06	49.02 ± 0.05
D_{max} (Å)	168	158	157	158	159
q range (Å^{-1})	0.006-0.3002	0.007-0.3002	0.0075-0.3002	0.007-0.3002	0.0075-0.3002
P(r) fit assessment (Quality estimate, χ^2)	0.8816, 1.211	0.8549, 1.2784	0.9476, 1.2327	0.948, 1.1474	0.8356, 1.1681

Antibody	SAP1.3				
Kabat	***h*IgG1**	**C232S+C233S**	**C233S κC214S**	**C232S κC214S**	***h*IgG2**
(b) Scattering particle size					
Methods/Software	BioXTAS RAW 2.3.0[18] for Porod volume and concentration-independent MW methods (Vc[20], MoW[21], Shape&Size[22], Bayesian inference[23]) Protparam (https://web.expasy.org/protparam/)[24] for MW from chemical composition				
Volume estimates (Å^3)					
Porod volume (ratio to M)	229000 (1.56)	222000 (1.51)	220000 (1.51)	213000 (1.45)	214000 (1.46)
Molecular weight (MW) estimates (Da)					
From chemical composition	147078	146571	146571	146571	146636
From concentration-independent method (kDa)					
Bayesian inference,	146.8	169.6	169.6	146.8	157.1
% confidence	50.9	66.9	88.8	67.4	64.1
(range, % confidence)	142.2-176.6, 94.1	151.4-176.6, 97.8	162.7-194.9, 96	134.3-162.7, 98.3	142.2-176.6,99.3
MoW	190.1	184.7	182.8	176.7	177.9
Vc	150.2	153.4	160.7	145.8	149.6
Size and shape	187.2	178.1	170.1	177	166.9

Supplementary Table 8: Crystallographic analysis of anti-*h*CD40 ChiLob7/4 *h*IgG2 F(ab')$_2$ variants.

Antibody	ChiLob7/4		
Kabat numbering	**cross-over + K228C**	**cross-over + T222C κE123C**	
Structure	C224S+K223C κC214S	C224S+T219C κE123C+κC214S	
PDB	8PUL	8PUK	
Data collection	DLS I23	ESRF ID30A-3	DLS I23
X-ray λ Å	2.7552	0.9677	2.7552
Space group	$P2_12_12_1$	P321	P321
Cell constants<br>a, b, c Å<br>α, β, γ °	74.99, 95.82, 151.93<br>90, 90, 90	148.17, 148.17, 45.30<br>90, 90, 120	147.06, 147.06, 45.037<br>90, 90, 120
Resolution Å[a]	151.92-1.93<br>(1.97-1.93)	74.19-2.67<br>(2.8-2.67)	127.36-2.67<br>(2.72-2.67)
Completeness %[a]	85.72 (73.7)	98.8 (97.1)	97.1 (93.3)
R_{pim}[a]	0.018 (0.729)	0.075 (0.986)	0.041 (0.377)
CC1/2[a]	0.997 (0.274)	0.995 (0.384)	0.997 (0.334)
I/sigma(I)[a]	16.93 (0.283)	10.1 (0.9)	9.0 (0.7)
Multiplicity[a]	9.58 (7.3)	7.1 (7.2)	16.3 (13.1)
Wilson B $Å^2$	50.2	70.6	57.4
Refinement			
Reflections (unique)	681254 (71110)	114834 (16178)	254448 (15644)
R, R_{free} %	20.3, 23.9	24.2, 28.2	
Average B $Å^2$	65.0	76.0	
RMSD bonds Å	0.0025	0.0047	
RMSD angles °	0.794	1.199	
Ramachandran # (%)<br>preferred, allowed, outlier	834 (97)<br>26 (3)<br>0 (0)	392 (94)<br>25 (6)<br>2 (0)	

[a] numbers in parentheses for high resolution bin

Supplementary Table 9: Binding affinity of anti-*h*CD40 ChiLob7/4 engineered disulfide variants. Surface plasmon resonance (SPR) was used to measure binding kinetics of engineered disulfide variants. Recombinant soluble *h*CD40-*h*Fc-His was immobilised on a CM5 sensor chip by amine coupling at 500 RU, and variants were injected over at a range of different concentrations (100, 20, 4, 0.8, 0.16 nM). **a** Table shows the k_a(association/on rate), k_d (dissociation/off rate) and K_D (k_d/k_a, Equilibrium dissociation constant). Values calculated from fitting sensorgrams to bivalent binding model and show mean ± range from 2 independent experiments. **b** Table shows Rmax and χ^2 values from the 2 experiments. Representative sensorgrams are shown in Supplementary Figure 17.

a

	**ChiLob7/4**		
	**k_a ($\times 10^4$ $M^{-1}s^{-1}$)**	**k_d ($\times 10^{-4}$ s^{-1})**	**K_D ($\times 10^{-9}$ M)**
***h*IgG1**	13.0 ± 0.70	0.747 ± 0.165	0.579 ± 0.159
**C232S κC214S (cross-over)**	6.18 ± 0.99	2.94 ± 0.670	4.74 ± 0.325
**cross-over + T222C κE123C**	12.1 ± 1.6	2.21 ± 0.310	1.82 ± 0.02
**cross-over + K228C**	12.6 ± 1.5	1.94 ± 0.340	1.52 ± 0.09
***h*IgG2**	8.33 ± 0.94	1.64 ± 0.0600	1.97 ± 0.151

b

	**ChiLob7/4**			
	Rmax exp1 (RU)	Rmax exp2 (RU)	χ^2 exp1	χ^2 exp2
***h*IgG1**	629.8	633	4.99	2.44
**C232S κC214S (cross-over)**	605.6	613.5	25.8	19.9
**cross-over + T222C κE123C**	646.6	652.8	10.8	7.04
**cross-over + K228C**	641	651.7	10.9	8.3
***h*IgG2**	607.4	647.3	23.1	20.9

Supplementary Table 10. Significance levels for *h*CD40 NF-κB GFP reporter assay. One-way ANOVA performed at each antibody concentration with Tukey's multiple comparisons test. n.s. $p > 0.05$,* $p < 0.05$, ** $p < 0.01$, *** $p < 0.001$, **** $p < 0.0001$. NF-κB GFP reporter assay dose response curve shown in Figure 4.

Concentration (μg/mL)	cross-over + K228C vs parental cross-over		cross-over + T222C κE123C vs parental cross-over	
5	***	p = 0.0004	**	p = 0.0018
1	*	p = 0.0271	n.s.	p = 0.0995
0.2	n.s.	p = 0.2019	n.s.	p = 0.4429
0.04	*	p = 0.0403	n.s.	p = 0.1142
0.008	**	p = 0.0015	**	p = 0.0017
0.0016	n.s.	p = 0.0946	n.s.	p = 0.1154
0.00032	n.s.	p = 0.4846	n.s.	p = 0.5857
0.000064	n.s.	p = 0.9928	n.s.	p = 0.9929

Supplementary Table 11. Significance levels for B cell assays. One-way ANOVA performed at each antibody concentration with Tukey's multiple comparisons test. n.s. $p > 0.05$,* $p < 0.05$, ** $p < 0.01$, *** $p < 0.001$, **** $p < 0.0001$. Graphs shown in Figure 4d-g.

Comparisons	HLA-DR		CD86		CD23		Proliferation	
*h*IgG1 vs 3	****	1.660E-05	****	1.474E-05	****	1.728E-07	n.s.	0.9166
*h*IgG1 vs 4	****	7.972E-09	****	5.786E-09	****	1.454E-10	**	0.0026
*h*IgG1 vs 5	****	2.343E-08	****	6.166E-09	****	1.337E-10	**	0.0042
*h*IgG1 vs *h*IgG2	****	5.65E-06	****	8.645E-06	****	6.884E-08	n.s.	0.0842
3 vs 4	****	2.924E-06	****	2.074E-06	****	6.328E-08	**	0.0087
3 vs 5	****	1.527E-05	****	2.263E-06	****	4.904E-08	*	0.0142
3 vs *h*IgG2	n.s.	0.7470	ns	0.9734	ns	0.4993	n.s.	0.2805
4 vs 5	n.s.	0.3679	ns	1.000	ns	0.9884	n.s.	0.9972
4 vs *h*IgG2	****	8.057E-06	****	3.314E-06	****	1.580E-07	n.s.	0.2213
5 vs *h*IgG2	****	4.980E-05	****	3.630E-06	****	1.211E-07	n.s.	0.3415

Supplementary Table 12. Significance levels for B cell assays (F(ab')$_2$). One-way ANOVA performed at each antibody concentration with Tukey's multiple comparisons test. n.s. p > 0.05,* p < 0.05, ** p < 0.01, *** p < 0.001, **** p < 0.0001. Graphs shown in Supplementary Figure 19.

Comparisons	HLA-DR		CD86		CD23	
*h*IgG1 vs 3	****	2.435E-05	****	2.425E-08	****	4.894E-08
*h*IgG1 vs 5	****	1.314E-06	****	2.130E-09	****	1.123E-09
*h*IgG1 vs *h*IgG2	****	2.606E-05	****	2.365E-08	****	1.525E-08
3 vs 5	**	0.0045	****	4.961E-06	****	3.087E-07
3 vs *h*IgG2	n.s.	0.9997	ns	0.9993	**	0.0043
5 vs *h*IgG2	**	0.0040	****	5.312E-06	****	3.961E-06

Supplementary Table 13: SAXS sample information, data collection and deposition (F(ab')$_2$).

(a) Sample details												
Antibody	ChiLob7/4							SAP1.3				
				cross-over	cross-over + K228C	cross-over + T222C κE123C						
Kabat	*h*IgG1	***h*IgG2 C232S+ C233S**	***h*IgG2 C233S κC214S**	***h*IgG2 C232S κC214S**	***h*IgG2 C232S+ K228C κC214S**	***h*IgG2 C232S+T222C κE123C+κC214S**	***h*IgG2**	*h*IgG1	***h*IgG2 C232S+ C233S**	***h*IgG2 C233S κC214S**	***h*IgG2 C232S κC214S**	***h*IgG2**
Structure	*h*IgG1	***h*IgG2 C224S+ C225S**	***h*IgG2 C225S κC214S**	***h*IgG2 C224S κC214S**	***h*IgG2 C224S+ K223C κC214S**	***h*IgG2 C224S+T219C κE123C+κC214S**	***h*IgG2**	*h*IgG1	***h*IgG2 C226S+ C227S**	***h*IgG2 C227S κC214S**	***h*IgG2 C226S κC214S**	***h*IgG2**
Organism	*Homo sapiens*											
Source	Recombinantly expressed in Chinese Hamster Ovary cells											
(b) Scattering particle composition												
Protein	Anti-*h*CD40 ChiLob7/4 antibody F(ab')$_2$ fragment with mutations as described.							Anti-*h*4-1BB SAP1.3 antibody F(ab')$_2$ fragment with mutations as described.				
Stoichiometry of components	n.a.							n.a.				
Solvent environment/configuration												
Solvent composition	50 mM HEPES, 150 mM KCl pH 7.5 (solvent blanks taken from SEC flowthrough prior to elution of protein)											
Sample temperature (˚C)	20											
In-beam sample cell	Quartz glass capillary, 1 mm diameter											

Antibody	ChiLob7/4							SAP1.3				
Kabat	*h*IgG1	*h*IgG2 C232S+ C233S	*h*IgG2 C233S κC214S	*h*IgG2 C232S κC214S	*h*IgG2 C232S+ K228C κC214S	*h*IgG2 C232S+T222C κE123C+κC214S	*h*IgG2	*h*IgG1	*h*IgG2 C232S+ C233S	*h*IgG2 C233S κC214S	*h*IgG2 C232S κC214S	*h*IgG2
(c) Size Exclusion Chromatography SEC-SAS												
Extinction coefficient [A_{280}, 0.1%(w/v)]	1.476	1.469	1.469	1.469	1.471	1.471	1.472	1.395	1.388	1.388	1.388	1.39
Sample injection concentration (mg/mL)	5.17	4.79	4.40	4.44	4.47	4.10	4.54	5.35	3.21	4.72	3.38	4.81
Sample injection volume (µL)	45											
SEC column type	Agilent Bio SEC-3 300 A pore size, 4.6 mm i.d., 300 mm length coupled to Shimadzu HPLC system											
SEC flowrate (mL/min)	0.3							0.2				
(d) SAS data collection												
Data acquisition/reduction software	ESRF SAXS Programs: data acquisition using *BSXCuBE3*, data reduction, data scaling and integration using PyFAI and analysis using FreeSAS[25]											
Source/instrument	ESRF SAXS beamline BM29 with Dectris PILATUS3 2M detector											
Measured *q*-range (q_{min} – q_{max}; nm^{-1})	0.025-6											
Exposure time (s), number of exposures	2 s x 490 frames							2 s x 750 frames				
Additional relevant details[g]	Wavelength 0.99 Å, beam size 200 µm x 200 µm, sample to detector distance 2.867 m											
e) Data and model deposition												
SASBDB IDs	SASDUG8	SASDUJ8	SASDUK8	SASDUL8	SASDSD7	SASDSC7	SASDUH8	SASDUF8	SASDUD8	SASDUC8	SASDUB8	SASDUE8

Supplementary Table 14: SAXS sample information, data collection and deposition (IgG).

(a) Sample details												
Antibody	ChiLob7/4							SAP1.3				
				cross-over	cross-over + K228C	cross-over + T222C κE123C						
Kabat	*h*IgG1	*h*IgG2 C232S+ C233S	*h*IgG2 C233S κC214S	*h*IgG2 C232S κC214S	*h*IgG2 C232S+ K228C κC214S	*h*IgG2 C232S+T222C κE123C+κC214S	*h*IgG2	*h*IgG1	*h*IgG2 C232S+ C233S	*h*IgG2 C233S κC214S	*h*IgG2 C232S κC214S	*h*IgG2
Structure	*h*IgG1	*h*IgG2 C224S+ C225S	*h*IgG2 C225S κC214S	*h*IgG2 C224S κC214S	*h*IgG2 C224S+ K223C κC214S	*h*IgG2 C224S+T219C κE123C+κC214S	*h*IgG2	*h*IgG1	*h*IgG2 C226S+ C227S	*h*IgG2 C227S κC214S	*h*IgG2 C226S κC214S	*h*IgG2
Organism	*Homo sapiens*											
Source	Recombinantly expressed in Chinese Hamster Ovary cells											
(b) Scattering particle composition												
Protein	Anti-*h*CD40 ChiLob7/4 antibody IgG with mutations as described.							Anti-*h*4-1BB SAP1.3 antibody IgG with mutations as described.				
Stoichiometry of components	n.a.							n.a.				
Solvent environment/configuration												
Solvent composition	50 mM HEPES, 150 mM KCl pH 7.5 (solvent blanks taken from SEC flowthrough prior to elution of protein)											
Sample temperature (˚C)	20											
In-beam sample cell	Quartz glass capillary, 1 mm diameter											

Antibody	ChiLob7/4							SAP1.3				
Kabat	*h*IgG1	***h*IgG2 C232S+ C233S**	***h*IgG2 C233S κC214S**	***h*IgG2 C232S κC214S**	***h*IgG2 C232S+ K228C κC214S**	***h*IgG2 C232S+T222C κE123C+κC214S**	***h*IgG2**	*h*IgG1	***h*IgG2 C232S+ C233S**	***h*IgG2 C233S κC214S**	***h*IgG2 C232S κC214S**	***h*IgG2**
(c) Size Exclusion Chromatography SEC-SAS												
Extinction coefficient [A $_{280}$, 0.1%(w/v)]	1.478	1.442	1.442	1.442	1.444	1.445	1.444	1.424	1.388	1.388	1.388	1.389
Sample injection concentration (mg/mL)	12.1	12.6	12.2	11.2	10.5	9.3	11.0	12.8	11.2	10.4	9.3	7.5
Sample injection volume (μL)	45 (for Lob7/4 *h*IgG2 C232S+C233S, 75 and for SAP1.3 *h*IgG2, 35)											
SEC column type	Agilent Bio SEC-3 300 A pore size, 4.6 mm i.d., 300 mm length coupled to Shimadzu HPLC system											
SEC flowrate (mL/min)	0.25											
(d) SAS data collection												
Data acquisition/reduction software	ESRF SAXS Programs: data acquisition using *BSXCuBE3*, data reduction, data scaling and integration using PyFAI and analysis using FreeSAS[25]											
Source/instrument	ESRF SAXS beamline BM29 with Dectris PILATUS3 2M detector											
Measured *q*-range (q_{min} – q_{max}; nm^{-1})	0.025-6											
Exposure time (s), number of exposures	2 s x 600 frames (for Lob7/4 *h*IgG2 C232S+C233S, 3 s x 500 frames)											
Additional relevant details[g]	Wavelength 0.99 Å, beam size 200 μm x 200 μm, sample to detector distance 2.867 m											
e) Data and model deposition												
SASBDB IDs	SASDWP2	SASDWU2	SASDWR2	SASDWQ2	SASDWS2	SASDWT2	SASDWN2	SASDWV2	SASDWZ2	SASDWY2	SASDWX2	SASDWW2

Supplementary Table 15: MD simulation set-up.

**MD simulation system set-up**	**Cross-over + K228C**	**Cross-over + T222C κE123C**
Simulation box dimensions	9.416 x 9.960 x 8.389 nm	10.187 x 9.725 x 7.773 nm
Total number of atoms	524405	480390
Total number of water molecules	169935	155290
Salt concentration	150 mM NaCl	150 mM NaCl
Lipid composition	N/A	N/A

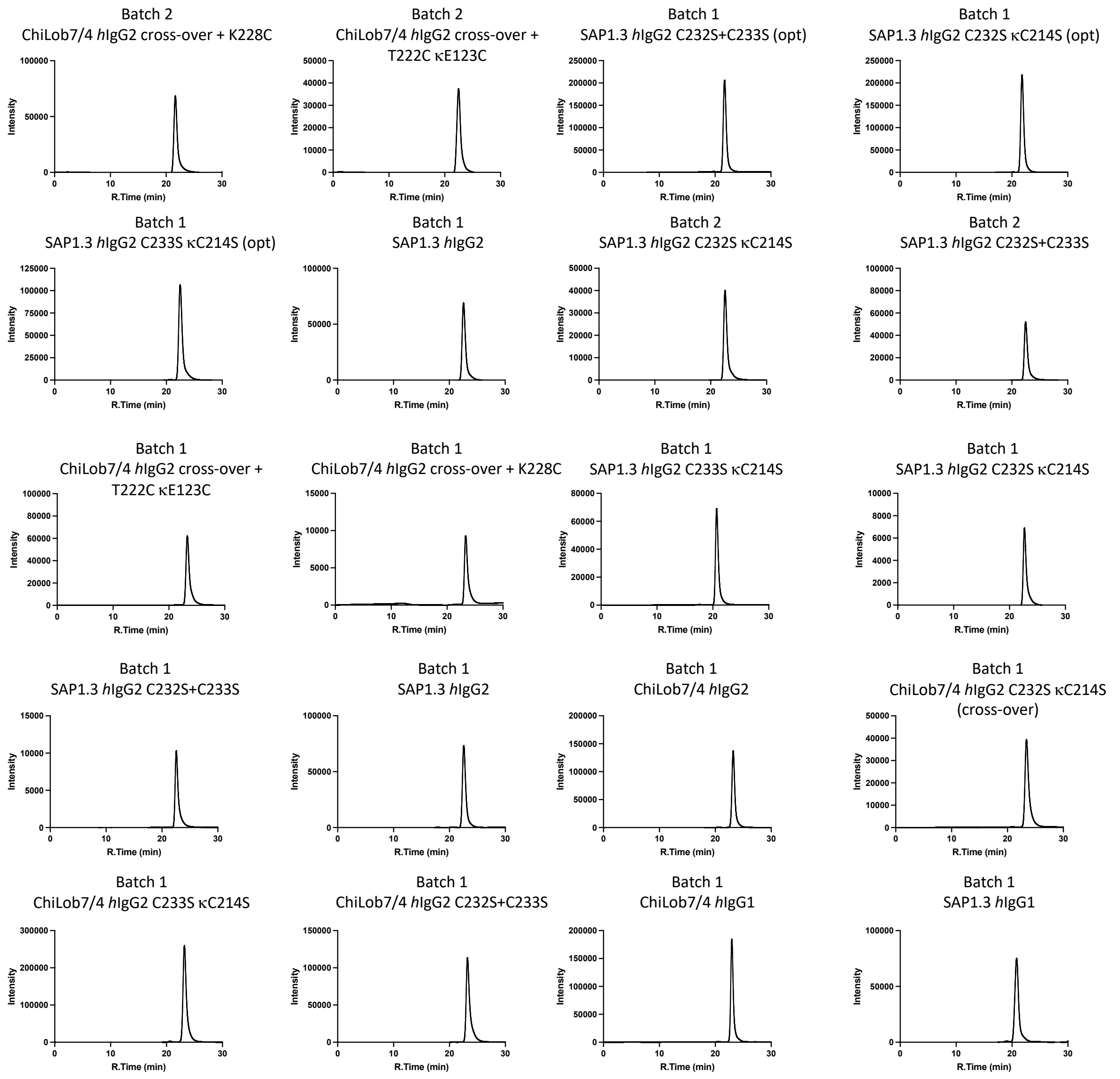

Supplementary Figure 1. HPLC traces of antibodies produced in-house. Antibody production details and final IgG purity over the aggregation peak are shown in Supplementary Table 1. Source data are provided as a Source Data file.

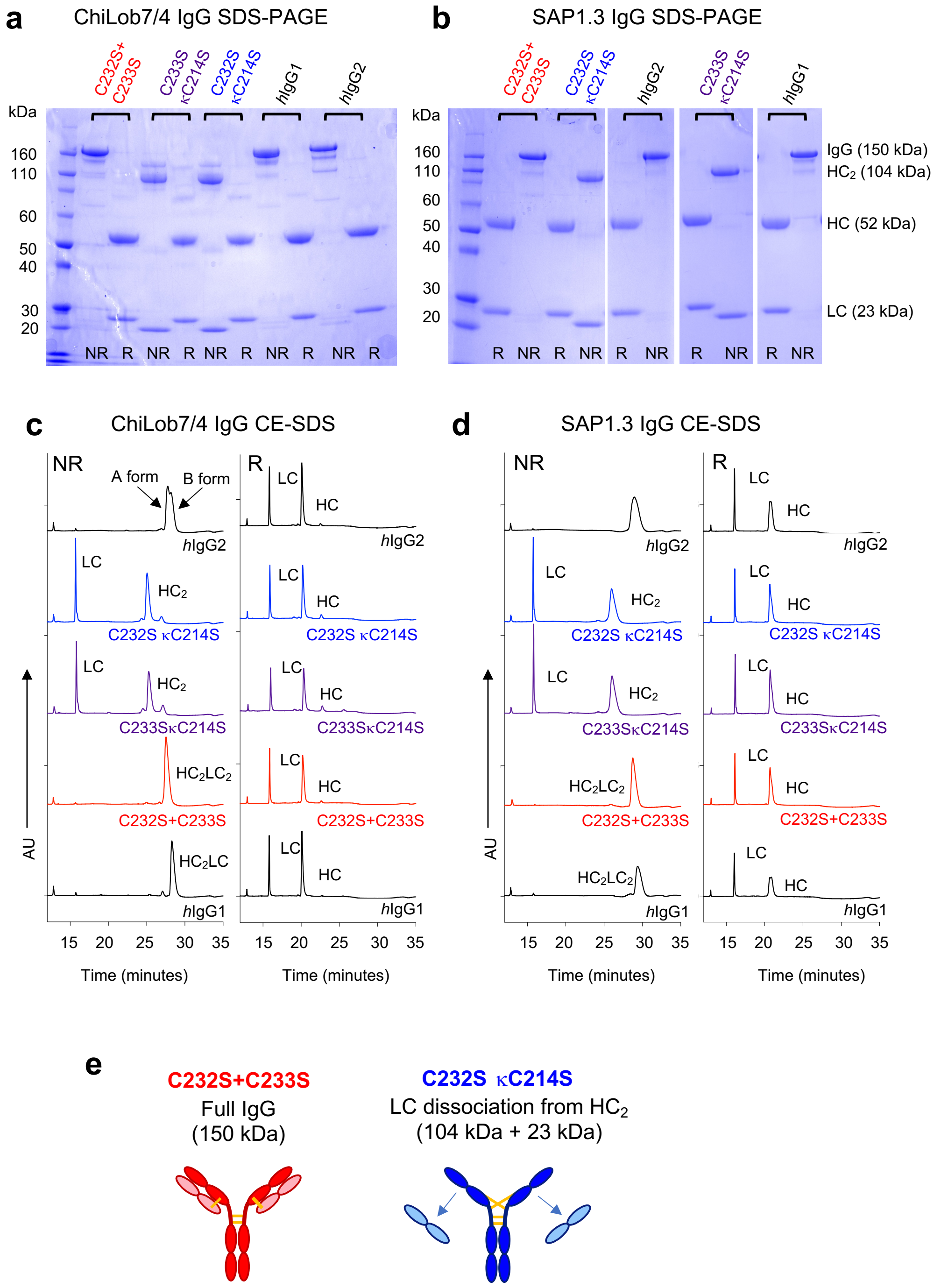

Supplementary Figure 2. Characterisation of anti-*h*CD40 ChiLob7/4 and anti-*h*4-1BB SAP1.3 mAb. IgG antibodies were analysed by **a, b** SDS-PAGE and **c, d** capillary electrophoresis with SDS (CE-SDS) under reducing (R) and non-reducing (NR) conditions. **e** shows a cartoon representation of a full IgG molecule for C232S+C233S and C232S κC214S whereby the light chains dissociate from the heavy chains of C232S κC214S due to the lack of stabilising disulfide bond (as depicted on the SDS-PAGE gels and CE-SDS electropherograms). HC = heavy chain, LC = light chain. HC_2 = heavy chain-heavy chain complex, HC_2LC_2 = heavy chain-heavy chain light chain-light chain complex, i.e. full IgG. *h*IgG1 and *h*IgG2 (black) shown as controls. Disulfide C-S antibody variants labelled by colour: red C232S+C233S, purple C233S κC214S, blue C232S κC214S. Source data are provided as a Source Data file.

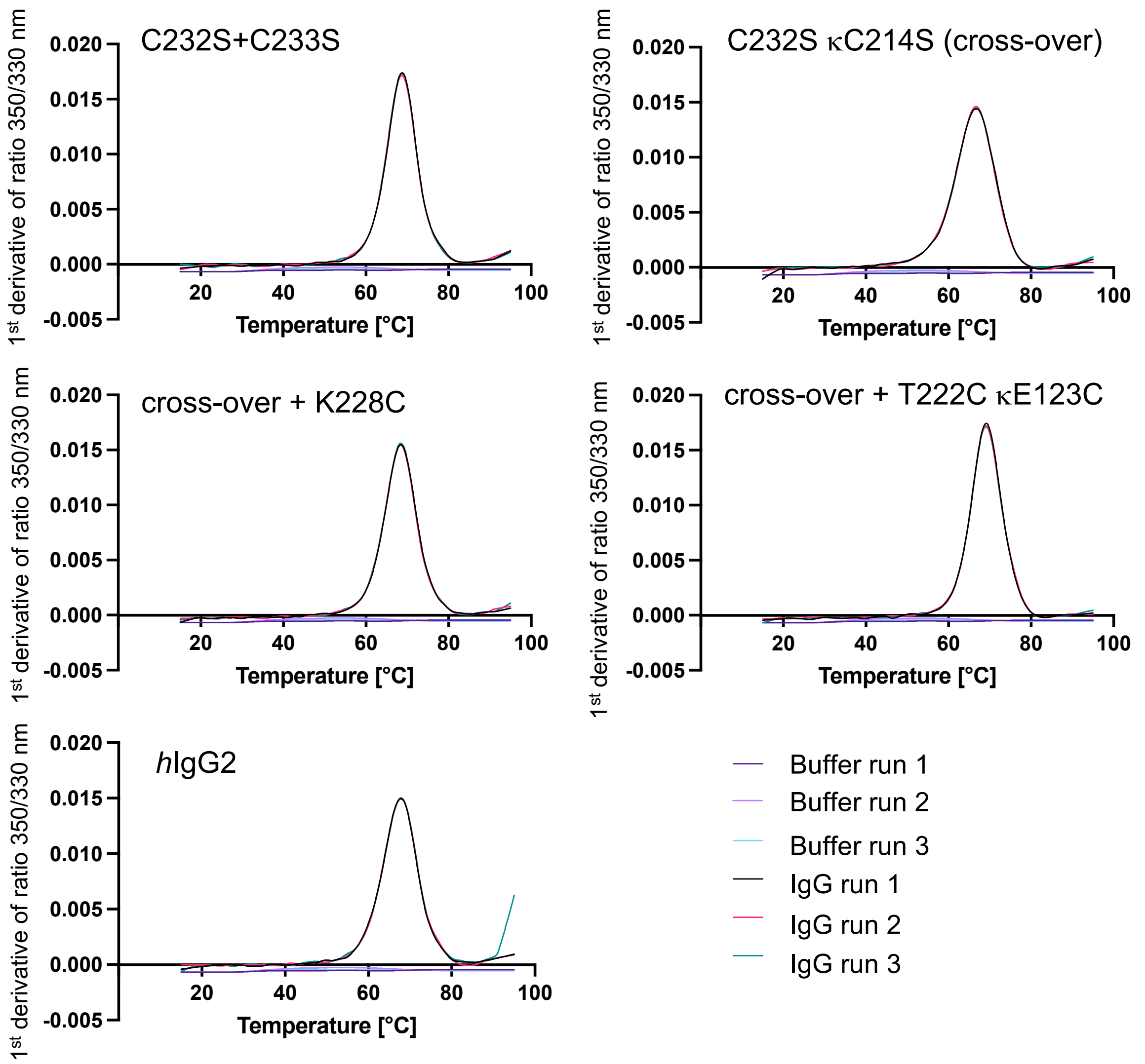

Supplementary Figure 3. Melting curves for anti-*h*CD40 ChiLob7/4 *h*IgG2 variants as IgG. NanoDSF was used to measure thermal stability of *h*IgG2 anti-*h*CD40 variants. Graphs show the first derivative of the ratio of 350 nm/ 330 nm for three repeats for each antibody. Melting temperatures reported in Supplementary Table 2 are the temperatures at the peak of the curves. Source data are provided as a Source Data file.

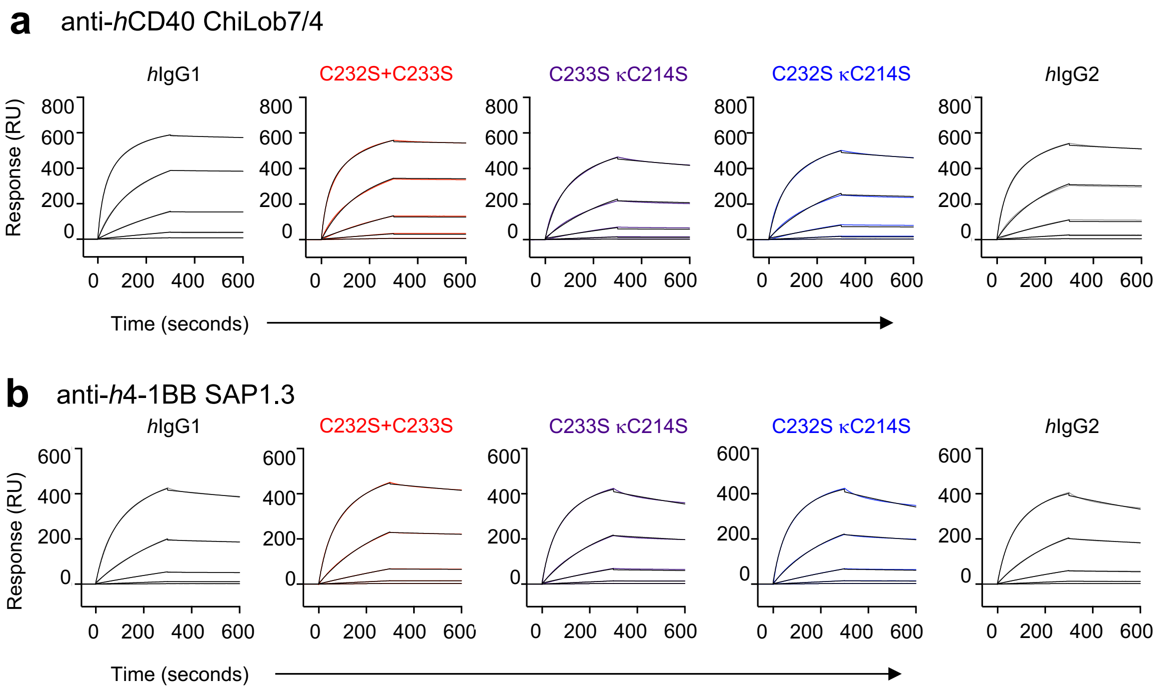

Supplementary Figure 4. Binding affinity of anti-*h*CD40 ChiLob7/4 and anti-*h*4-1BB SAP1.3 IgG variants. Surface plasmon resonance (SPR) was used to measure binding kinetics of variants. Recombinant soluble *h*CD40-*h*Fc-His or *h*4-1BB-*h*Fc-His was immobilised on a CM5 sensor chip by amine coupling at 500 RU, and hinge variants were injected over at a range of different concentrations (100, 20, 4, 0.8, 0.16 nM) at 25 °C. Graphs show representative sensorgrams at the 5 concentrations for each of the variants for n=2, **a** shows anti-*h*CD40 ChiLob7/4 antibodies and **b** shows anti-*h*4-1BB SAP1.3 antibodies. Experimental curves shown in colour with bivalent fitted curves shown in black. *h*IgG1 and *h*IgG2 (grey) shown as controls. Disulfide C-S antibody variants labelled by colour: red C232S+C233S, purple C233S κC214S, blue C232S κC214S. Affinity constants shown in Supplementary Table 3. Source data are provided as a Source Data file.

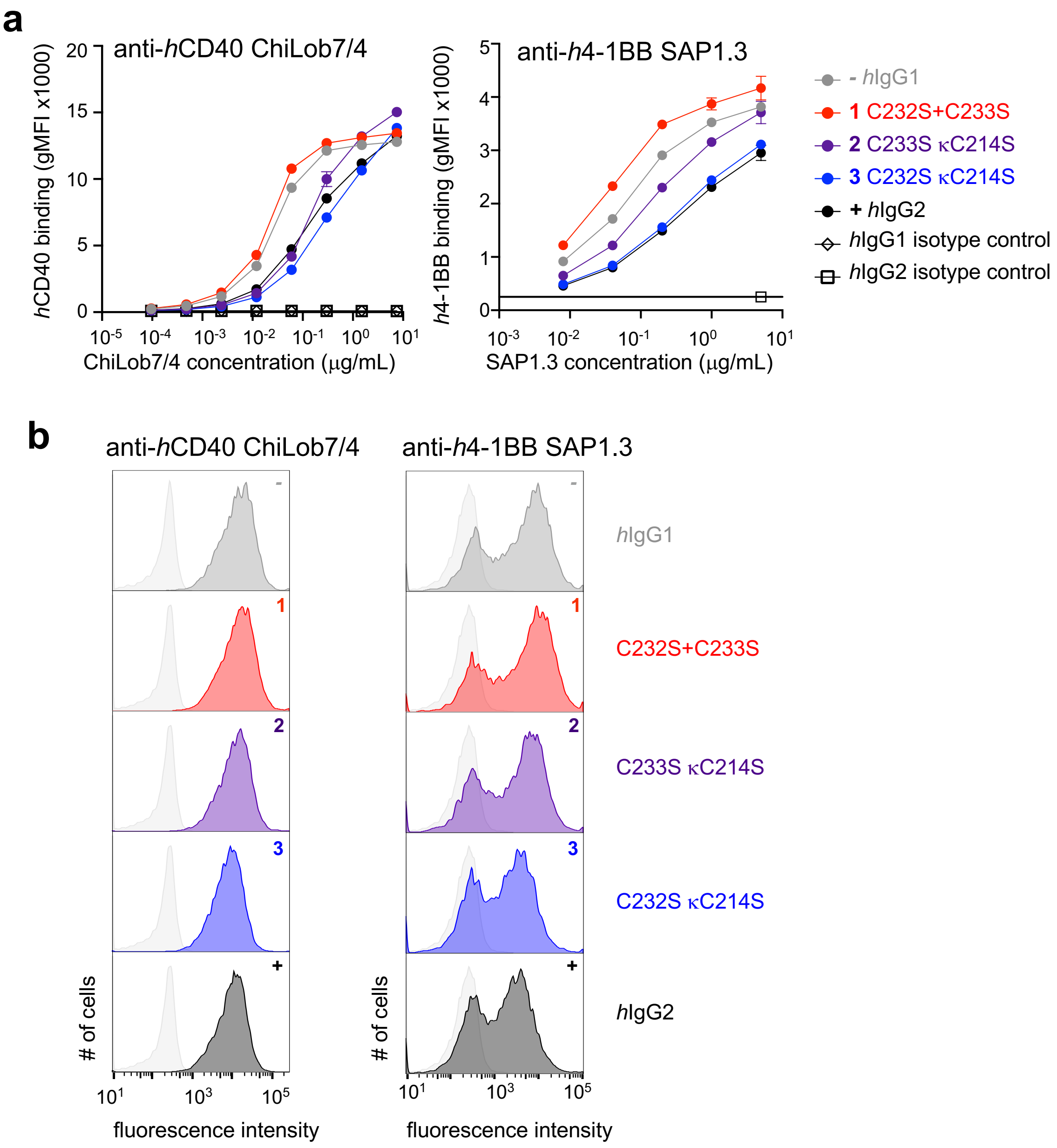

Supplementary Figure 5. Receptor binding for anti-*h*CD40 and anti-*h*4-1BB IgG variants. a Serially diluted ChiLob7/4 or SAP1.3 mAb variants were incubated with Jurkat cells expressing either *h*CD40 or *h*41BB, respectively, for 30 mins at 4 °C. Binding was detected by flow cytometry with a secondary PE-conjugated polyclonal goat F(ab')$_2$ anti-*h*Fc. Graphs show binding dose response curves of geometric mean fluorescence intensity (gMFI). Mean ± SEM, n = 2 independent experiments, mean taken from technical triplicate for each independent experiment. **b** Representative flow cytometry histogram overlays at a concentration of 0.3 μg/mL (for ChiLob7/4) or 0.2 μg/mL (for SAP1.3). *h*IgG1 (grey) and *h*IgG2 (black) shown as controls. Disulfide C-S antibody variants labelled by colour: red C232S+C233S, purple C233S κC214S, blue C232S κC214S. Source data are provided as a Source Data file.

anti-*h*CD40 ChiLob7/4 F(ab')$_2$

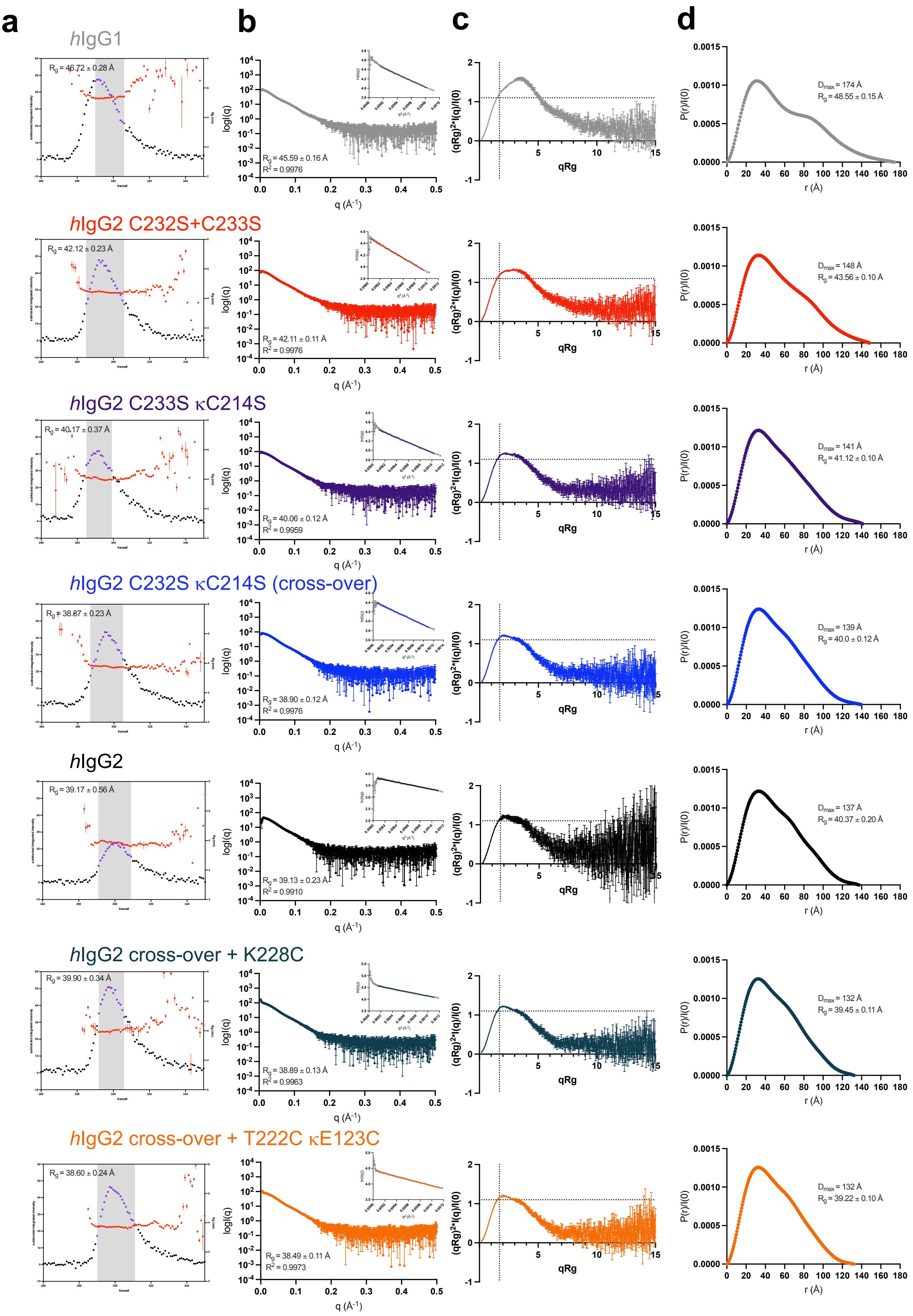

Supplementary Figure 6. SAXS analysis for anti-*h*CD40 ChiLob7/4 F(ab')$_2$ variants. a SEC-SAXS traces showing intensity (black circles) and Rg (red circles) as a function of frame number. Data frames coloured purple (shown within shaded grey region) were selected for averaging to obtain I(q) vs. q. Average R_g across the SEC elution peak per subtracted frame ± SD is shown inset. **b** I(q) vs q shown as log-linear plots with the inset showing the Guinier fits (coloured line) for $qR_g < 1.3$. Guinier R_g shown inset. **c** Dimensionless Kratky plots for the data in b. **d** P(r) vs r profiles for the data in b. D_{max} and R_g calculated from the P(r) distribution shown inset. SAXS data table for ChiLob7/4 F(ab')$_2$ variants shown in Supplementary Table 4. *h*IgG1 (grey) and *h*IgG2 (black) shown as controls. Engineered antibody variants labelled by colour: red C232S+C233S, purple C233S κC214S, blue C232S κC214S, teal cross-over + K228C, orange cross-over + T222C κE123C. Source data are provided as a Source Data file.

anti-*h*4-1BB SAP1.3 F(ab')$_2$

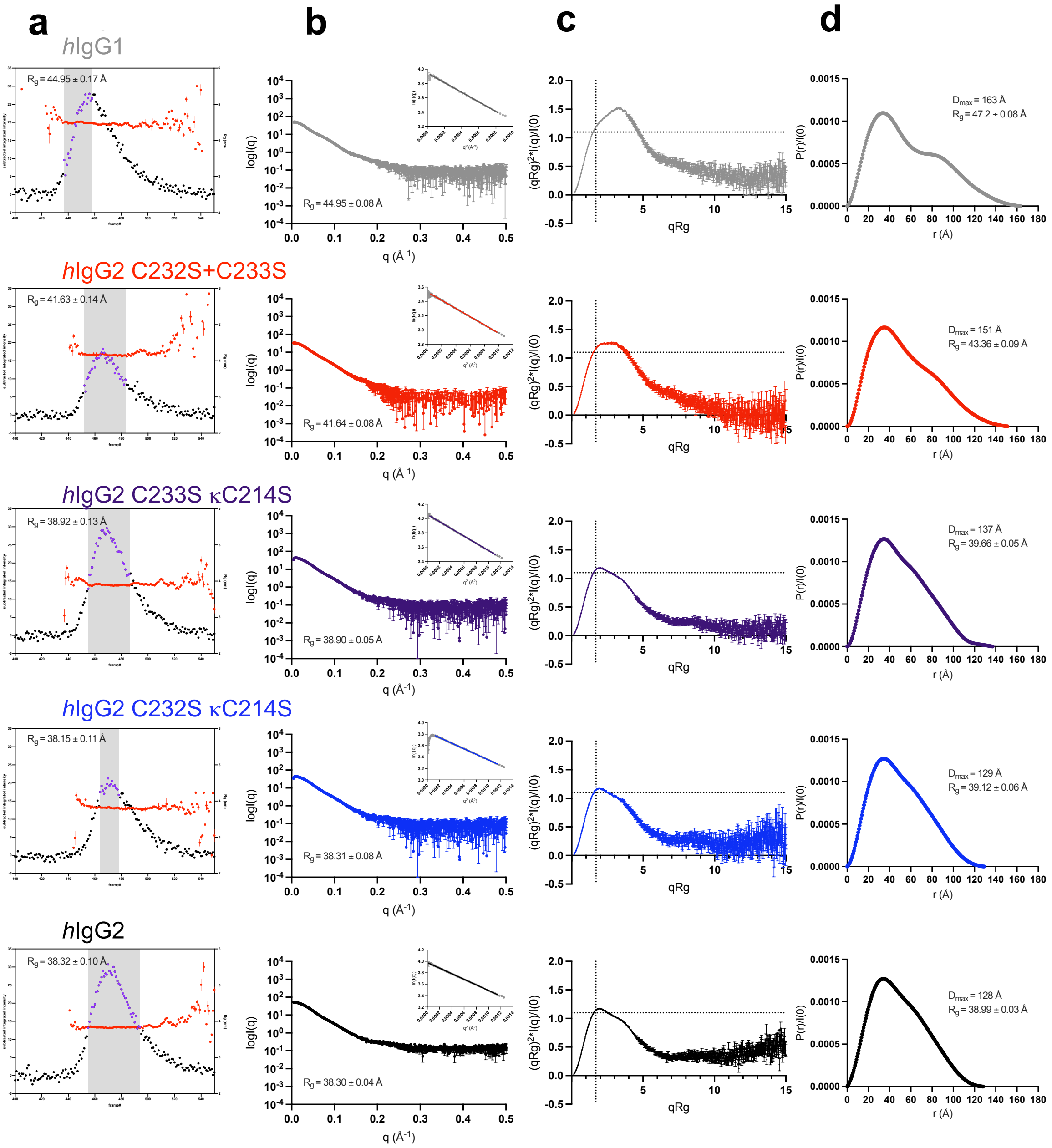

Supplementary Figure 7. SAXS analysis for anti-*h*4-1BB SAP1.3 F(ab')$_2$ variants. a SEC-SAXS traces showing intensity (black circles) and Rg (red circles) as a function of frame number. Data frames coloured purple (shown within shaded grey region) were selected for averaging to obtain I(q) vs. q. Average R_g across the SEC elution peak per subtracted frame ± SD is shown inset. **b** I(q) vs q shown as log-linear plots with the inset showing the Guinier fits (coloured line) for $qR_g < 1.3$. Guinier R_g shown inset. **c** Dimensionless Kratky plots for the data in b. **d** P(r) vs r profiles for the data in b. D_{max} and R_g calculated from the P(r) distribution shown inset. SAXS data table for SAP1.3 F(ab')$_2$ variants shown in Supplementary Table 5. *h*IgG1 (grey) and *h*IgG2 (black) shown as controls. Engineered antibody variants labelled by colour: red C232S+C233S, purple C233S κC214S, blue C232S κC214S. Source data are provided as a Source Data file.

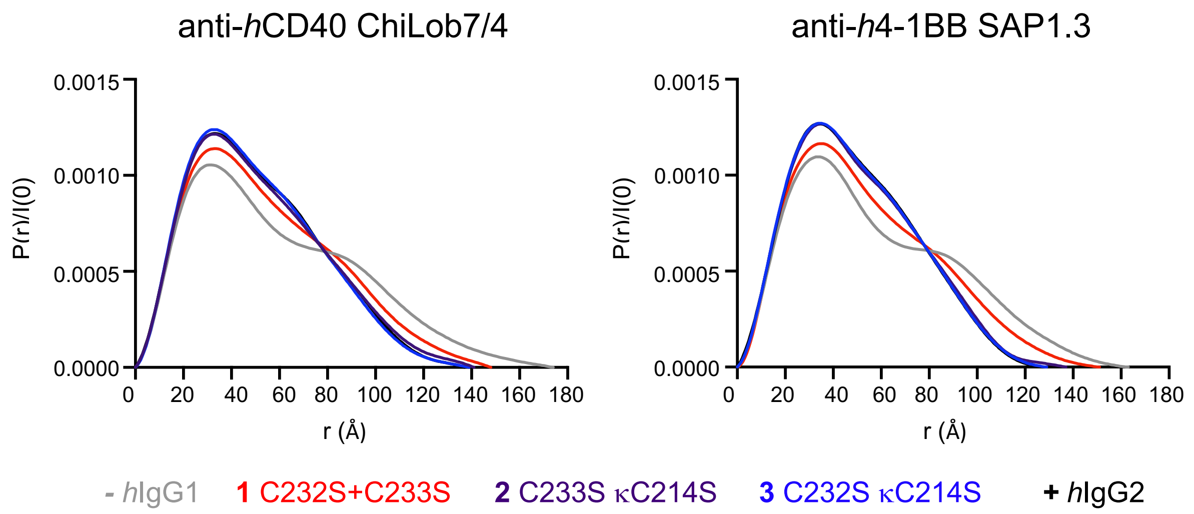

Supplementary Figure 8. P(r) distribution overlays for anti-*h*CD40 and anti-*h*4-1BB F(ab')$_2$ variants. *h*IgG1 (grey) and *h*IgG2 (black) shown as controls. Disulfide C-S antibody variants labelled by colour: red C232S+C233S, purple C233S κC214S, blue C232S κC214S. Source data are provided as a Source Data file.

anti-*h*CD40 ChiLob7/4 IgG

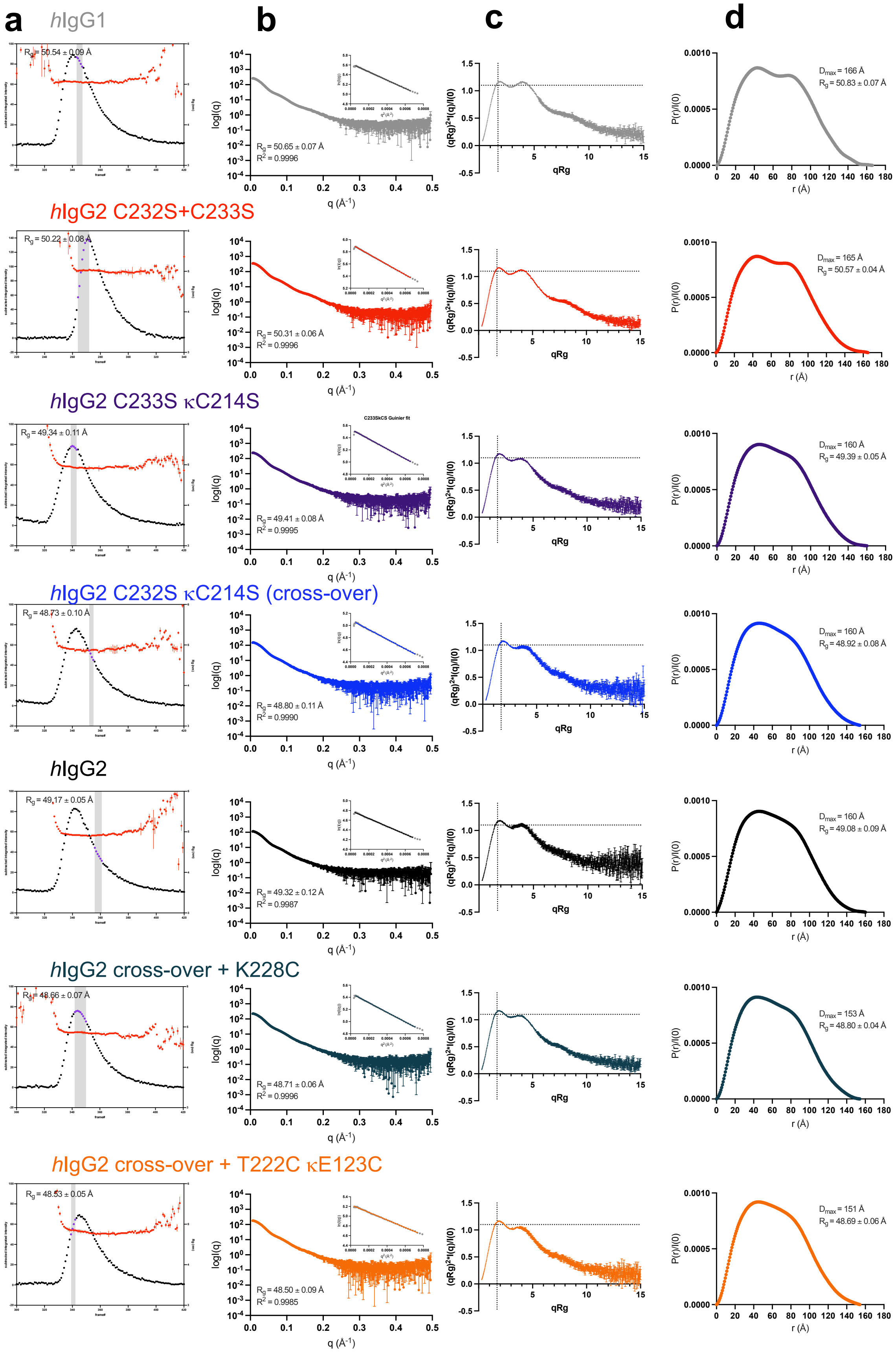

Supplementary Figure 9. SAXS analysis for anti-*h*CD40 ChiLob7/4 IgG variants. a SEC-SAXS traces showing intensity (black circles) and Rg (red circles) as a function of frame number. Data frames coloured purple (shown within shaded grey region) were selected for averaging to obtain I(q) vs. q. Average R_g across the SEC elution peak per subtracted frame ± SD is shown inset. **b** I(q) vs q shown as log-linear plots with the inset showing the Guinier fits (coloured line) for $qR_g < 1.3$. Guinier R_g shown inset. **c** Dimensionless Kratky plots for the data in b. **d** P(r) vs r profiles for the data in b. D_{max} and R_g calculated from the P(r) distribution shown inset. SAXS data table for ChiLob7/4 IgG variants shown in Supplementary Table 6. *h*IgG1 (grey) and *h*IgG2 (black) shown as controls. Engineered antibody variants labelled by colour: red C232S+C233S, purple C233S κC214S, blue C232S κC214S, teal cross-over + K228C, orange cross-over + T222C κE123C. Source data are provided as a Source Data file.

anti-*h*4-1BB SAP1.3 IgG

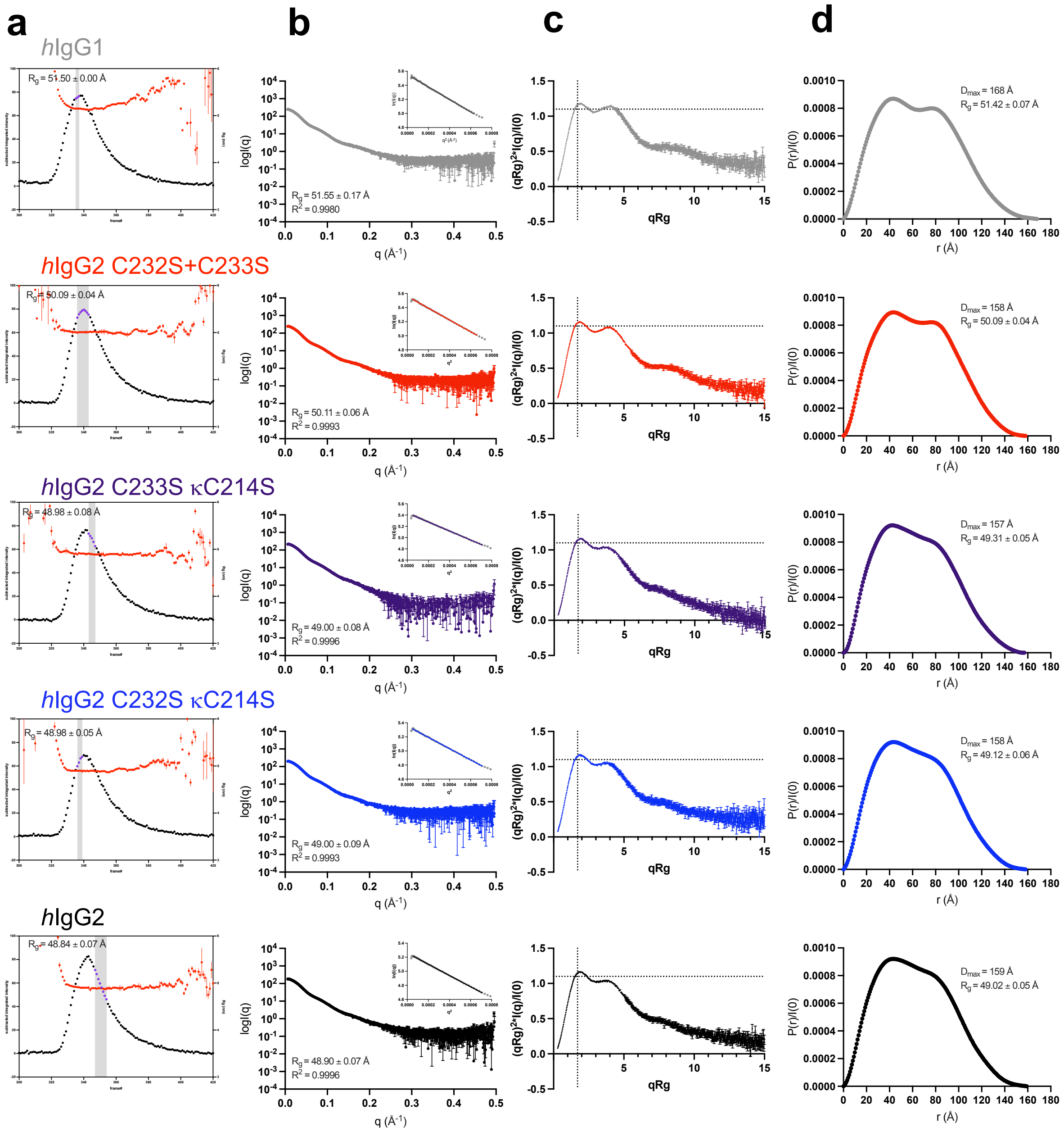

Supplementary Figure 10. SAXS analysis for anti-*h*4-1BB SAP1.3 IgG variants. a SEC-SAXS traces showing intensity (black circles) and Rg (red circles) as a function of frame number. Data frames coloured purple (shown within shaded grey region) were selected for averaging to obtain I(q) vs. q. Average R_g across the SEC elution peak per subtracted frame ± SD is shown inset. **b** I(q) vs q shown as log-linear plots with the inset showing the Guinier fits (coloured line) for $qR_g < 1.3$. Guinier R_g shown inset. **c** Dimensionless Kratky plots for the data in b. **d** P(r) vs r profiles for the data in b. D_{max} and R_g calculated from the P(r) distribution shown inset. SAXS data table for SAP1.3 IgG variants shown in Supplementary Table 7. *h*IgG1 (grey) and *h*IgG2 (black) shown as controls. Engineered antibody variants labelled by colour: red C232S+C233S, purple C233S κC214S, blue C232S κC214S. Source data are provided as a Source Data file.

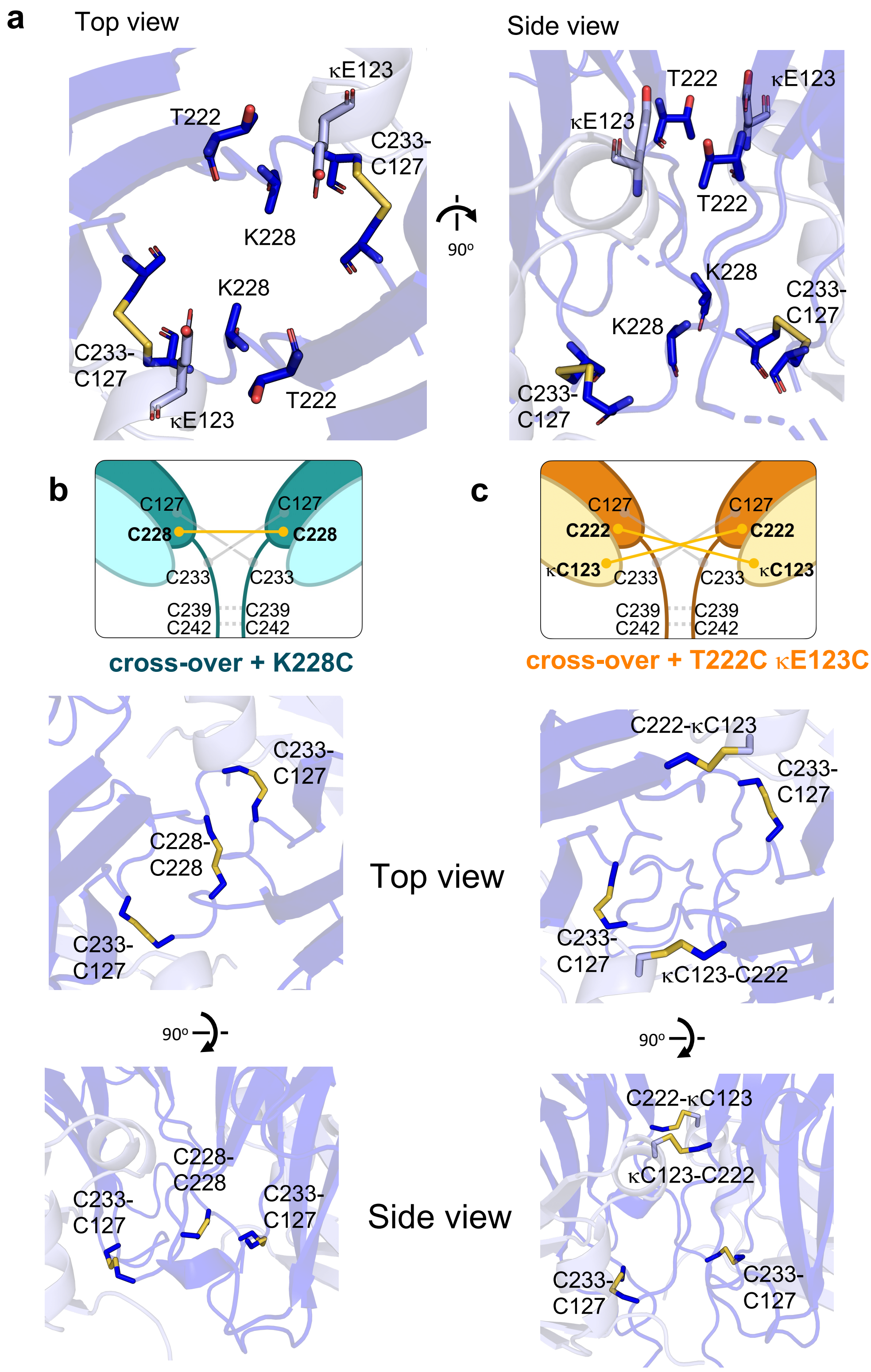

Supplementary Figure 11. Structure-guided design of engineered disulfide variants. a Cartoon representation of the cross-over variant C232S κC214S (PDB ID: 6TKE) showing residues of interest as sticks. Heavy chain coloured dark blue, light chain coloured light blue. Disulfides in yellow. **b** Predicted disulfide pattern for the cross-over + K228C variant. **c** Predicted disulfide pattern for the cross-over + T222C κE123C variant. Engineered antibody variants labelled by colour: teal cross-over + K228C, orange cross-over + T222C κE123C.

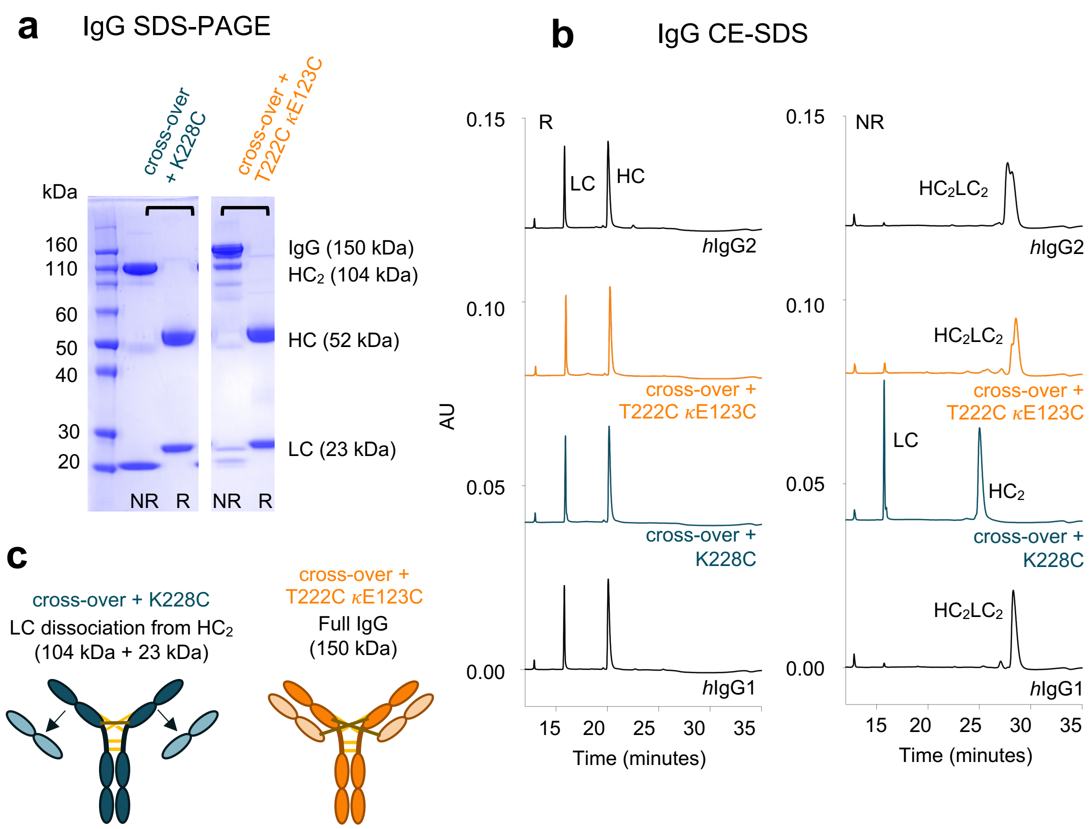

Supplementary Figure 12. Characterisation of anti-*h*CD40 ChiLob7/4 engineered disulfide variants. IgG antibodies were produced and analysed under reducing (R) and non-reducing (NR) conditions by **a** SDS-PAGE and **b** CE-SDS. HC = heavy chain, LC = light chain. HC_2 = heavy chain-heavy chain complex, HC_2LC_2 = heavy chain-heavy chain light chain-light chain complex i.e. full IgG. Compare Supplementary Figure 1. **c** Model explaining how in the cross-over + K228C variant the light chains can dissociate from the heavy chains due to the lack of stabilising disulfide bond, while in the cross-over + T222C κE123C variant this is not the case if a second pair of disulfides forms to link opposing light and heavy chains. *h*IgG1 and *h*IgG2 (black) shown as controls. Engineered antibody variants labelled by colour: teal cross-over + K228C, orange cross-over + T222C κE123C. Source data are provided as a Source Data file.

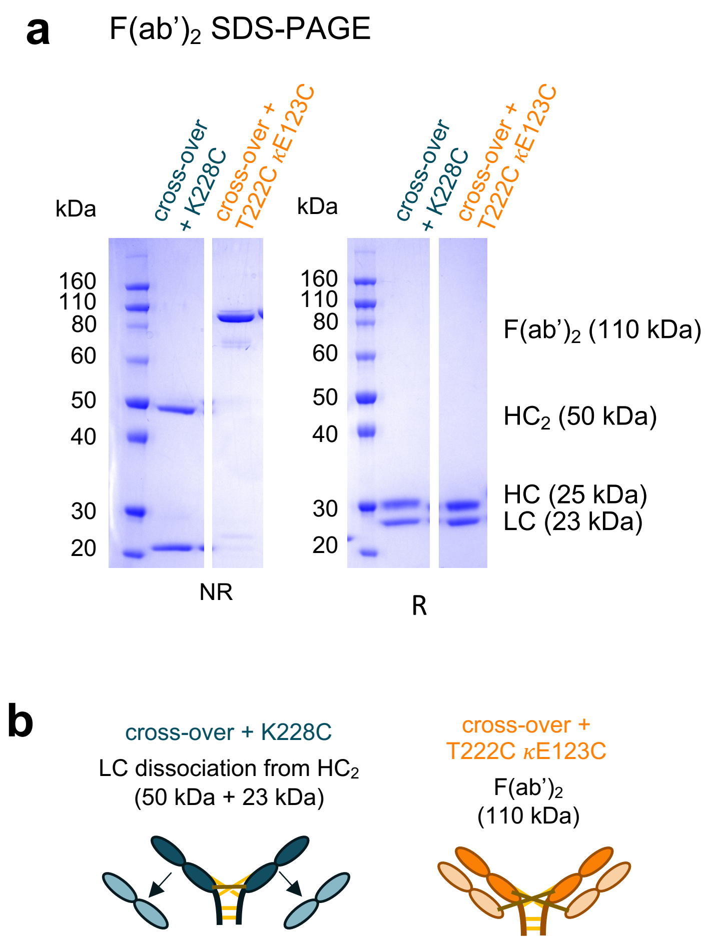

Supplementary Figure 13. Characterisation of anti-*h*CD40 ChiLob7/4 engineered disulfide variants as F(ab')$_2$ fragments. a F(ab')$_2$ fragments were produced and characterised by SDS-PAGE under reducing (R) and non-reducing (NR) conditions for the cross-over + K228C and the cross-over + T222C κE123C variant. HC = heavy chain, LC = light chain. HC$_2$ = heavy chain-heavy chain complex. **b** Model explaining how in the cross-over + K228C variant the light chains can dissociate from the heavy chains due to the lack of stabilising disulfide bond, while in the cross-over + T222C κE123C variant this is not the case if a second pair of disulfides forms to link opposing light and heavy chains. Compare Supplementary Figure 13 for whole IgG. Engineered antibody variants labelled by colour: teal cross-over + K228C, orange cross-over + T222C κE123C. Source data are provided as a Source Data file.

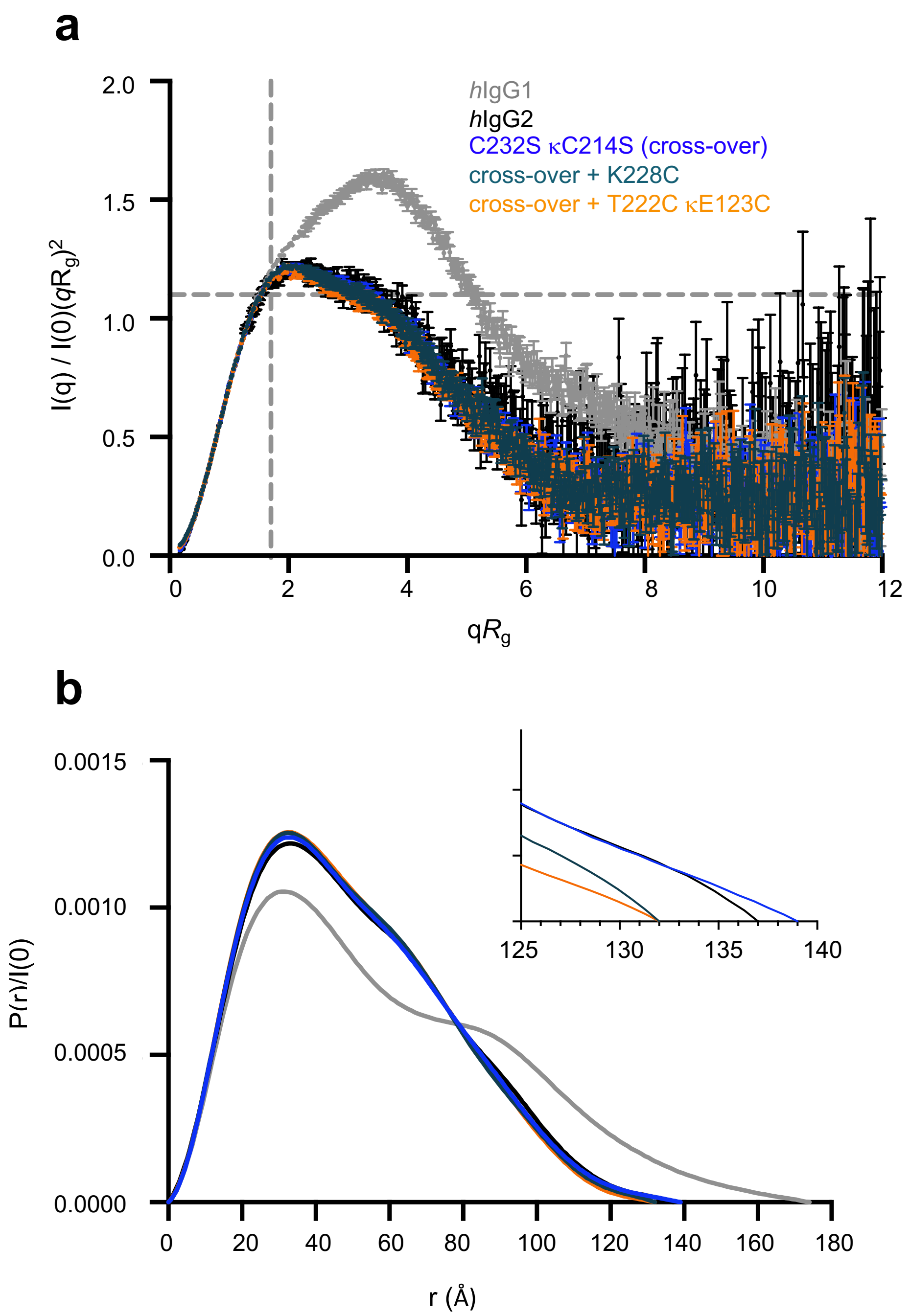

Supplementary Figure 14. SAXS analysis of anti-*h*CD40 ChiLob7/4 engineered disulfide variants as F(ab')$_2$ fragments. F(ab')$_2$ fragments of anti-*h*CD40 engineered disulfide variants were analysed by SEC-SAXS. **a** Graph shows an overlay of dimensionless Kratky plots, with the Guinier-Kratky point (√3, 1.103) shown by the crosshairs. **b** Graph shows an overlay of P(r) distributions with a zoomed in overlay of the x axis, top right inset. *h*IgG1 (grey) and *h*IgG2 (black) shown as controls. Engineered antibody variants labelled by colour: blue C232S κC214S, teal cross-over + K228C, orange cross-over + T222C κE123C. Source data are provided as a Source Data file.

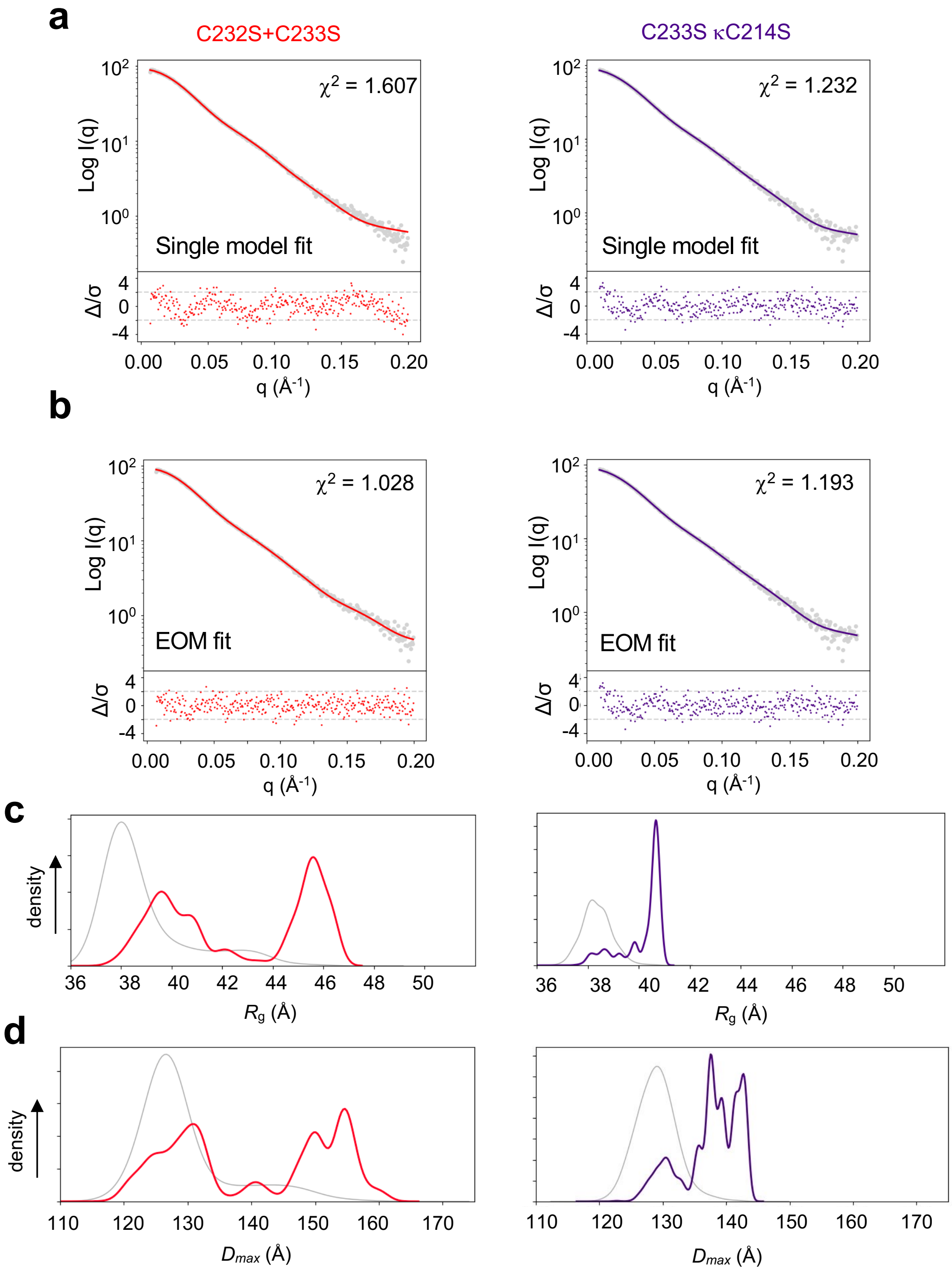

Supplementary Figure 15. Ensemble optimisation analysis for ChiLob7/4 variants as F(ab')$_2$ fragments. Ensemble optimisation of MD-generated conformational pools was performed using GAJOE for variants where fits to single models extracted from the MD pool were poor. Models were extracted every 1 ns from 6 μs of MD simulation for each F(ab')$_2$. **a** Agreement of the calculated scattering curve from the best fitting single model from the MD conformation pool (colour) with experimental SAXS data (grey dots) as indicated by the χ^2 fit with error-weighted residuals plot below. **b** Agreement of the calculated scattering curve from the reweighted ensemble (colour) with experimental SAXS data (grey dots) as indicated by the χ^2 fit with error-weighted residuals plot below, EOM refers to ensemble optimisation method (GAJOE). Graphs show the distribution of structures plotted **c** against R_g and **d** against D_{max} for the MD-generated starting conformational pool (shown in grey) and the reweighted selected ensemble (shown in colour). Engineered antibody variants labelled by colour: red C232S+C233S, purple C233S κC214S. Source data are provided as a Source Data file.

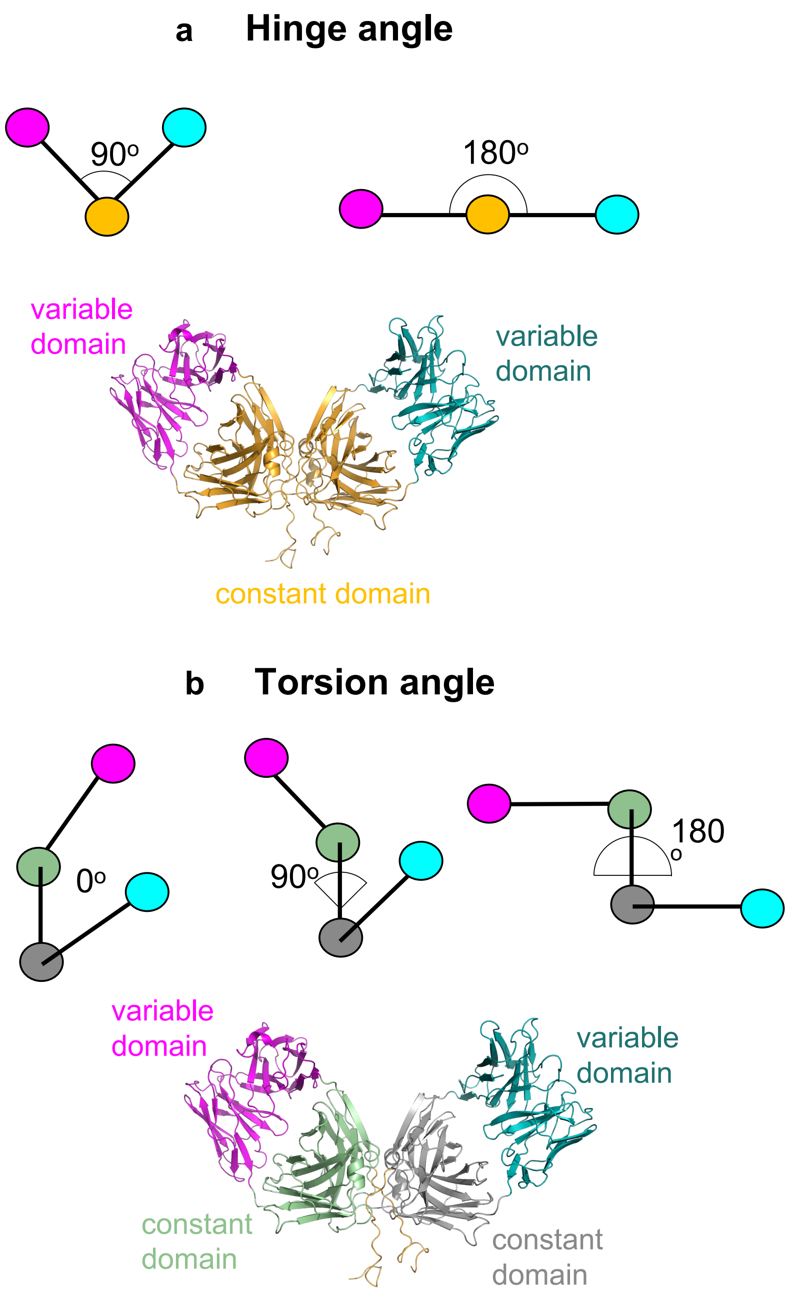

Supplementary Figure 16. Cartoon representation of the calculation of hinge and torsion angles for antibody F(ab')$_2$ molecules. a The hinge angle is calculated as the angle between the centre of mass (COM) of the variable domain of one F(ab') arm, the COM of the constant domain of both F(ab') arms (including the hinge region) and the COM of the variable domain of the second F(ab') arm. **b** The torsion angle is calculated as the dihedral angle between the COM of the variable domain of one F(ab') arm, the COM of the constant domain of that F(ab') arm, the COM of the constant domain of the second F(ab') arm and the COM of the variable domain of the second F(ab') arm. See Methods for further details.

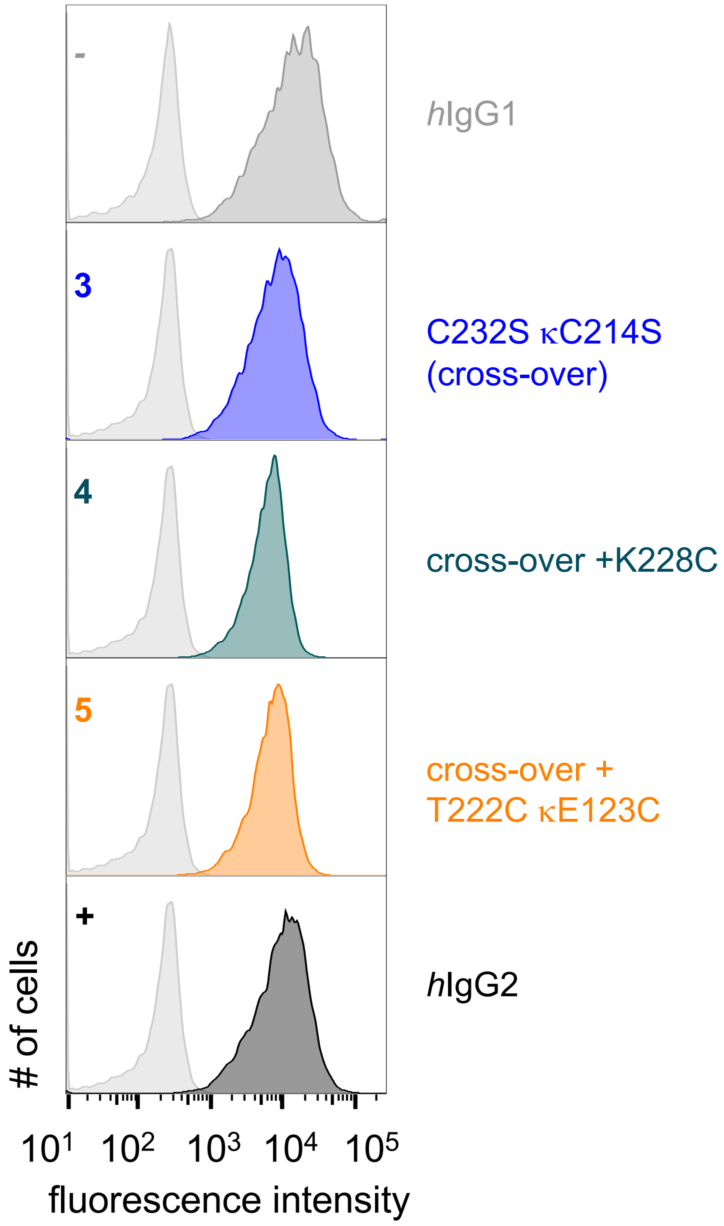

Supplementary Figure 17. Cell surface receptor binding of ChiLob7/4 IgG engineered disulfide variants. Serially diluted ChiLob7/4 variants were incubated with Jurkat cells expressing *h*CD40 with binding detected with a secondary PE-conjugated antibody by flow cytometry. Representative flow cytometry histogram overlays at a concentration of 0.3 μg/mL. In pale grey is the *h*IgG1 or *h*IgG2 isotype control, as appropriate. *h*IgG1 (grey) and *h*IgG2 (black) shown as controls. Engineered antibody variants labelled by colour: blue C232S κC214S, teal cross-over + K228C, orange cross-over + T222C κE123C. Source data are provided as a Source Data file.

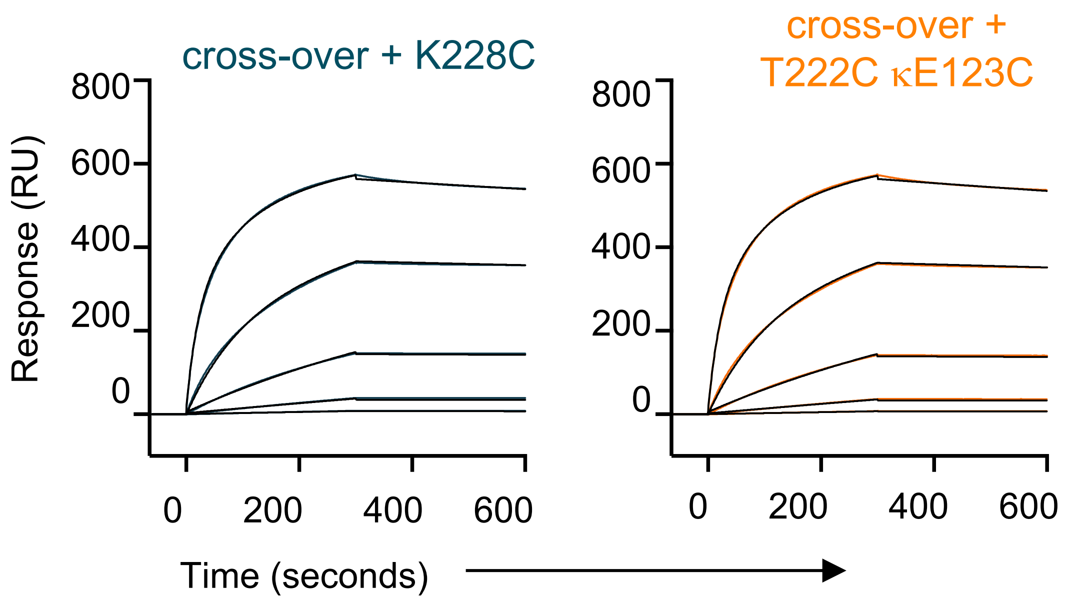

Supplementary Figure 18. Binding affinity of ChiLob7/4 IgG engineered disulfide variants. SPR was used to measure binding kinetics of engineered disulfide variants. Recombinant soluble *h*CD40-*h*Fc-His was immobilised on an CM5 sensor chip by amine coupling at 500 RU, and variants were injected over at a range of different concentrations (100, 20, 4, 0.8, 0.16 nM). Graphs show representative sensorgrams for the 5 concentrations for each of the variants from n=2. Experimental curves shown in colour with bivalent fitted curves shown in black. Affinity constants shown in Supplementary Table 9. Engineered antibody variants labelled by colour: teal cross-over + K228C, orange cross-over + T222C κE123C. Source data are provided as a Source Data file.

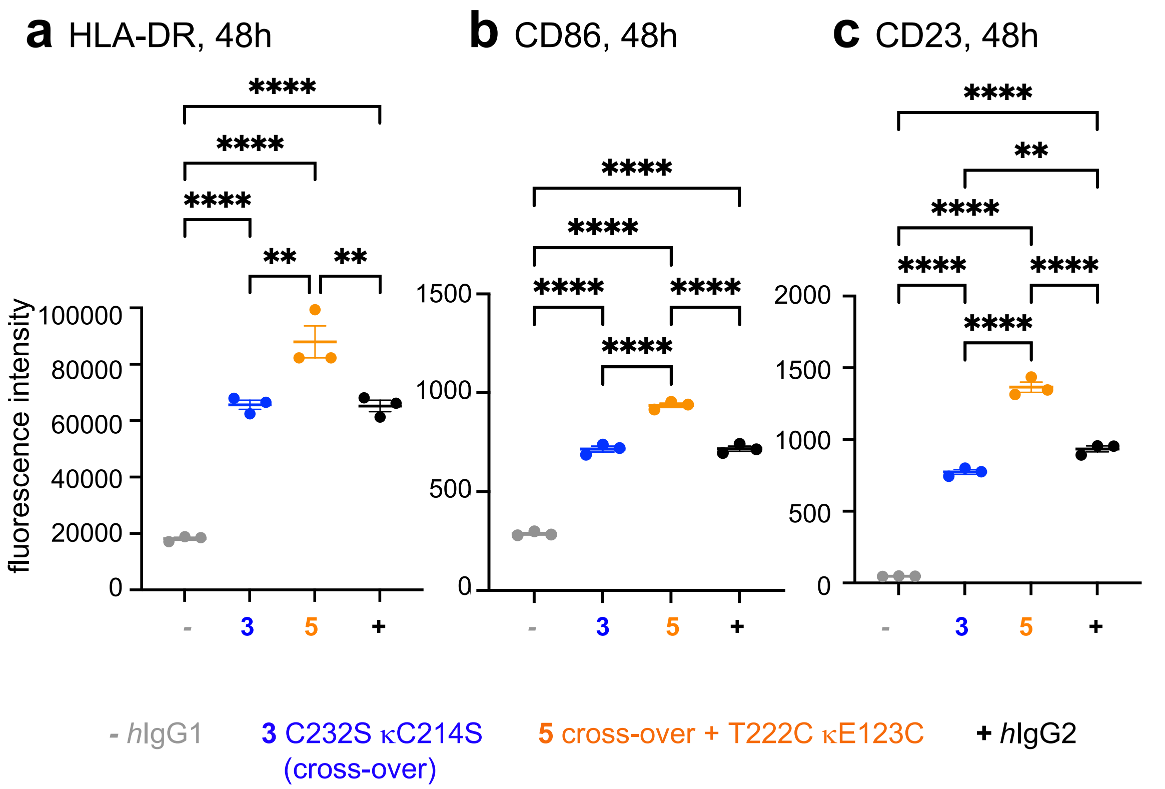

Supplementary Figure 19. Agonistic activity of engineered anti-*h*CD40 ChiLob7/4 F(ab')$_2$ fragments. ChiLob7/4 F(ab')$_2$ variants were incubated with human B cells and activation assays performed. Activation of primary human B cells determined by upregulation of HLA-DR (**a**), CD86 (**b**), CD23 (**c**); measurements taken 48h after addition of 0.008 μg/mL ChiLob7/4 F(ab')$_2$. * $p < 0.05$, ** $p < 0.01$, *** $p < 0.001$, **** $p < 0.0001$ one-way ANOVA with Tukey's multiple comparisons test (for exact p-values, see Supplementary Table 12). Data show technical triplicates from 1 of 3 independent experiments with independent donors. Compare Supplementary Figure 4 for the same assays using whole IgG rather than F(ab')$_2$. *h*IgG1 (grey) and *h*IgG2 (black) shown as controls. Engineered antibody variants labelled by colour: blue C232S κC214S, teal cross-over + K228C, orange cross-over + T222C κE123C. Source data are provided as a Source Data file.

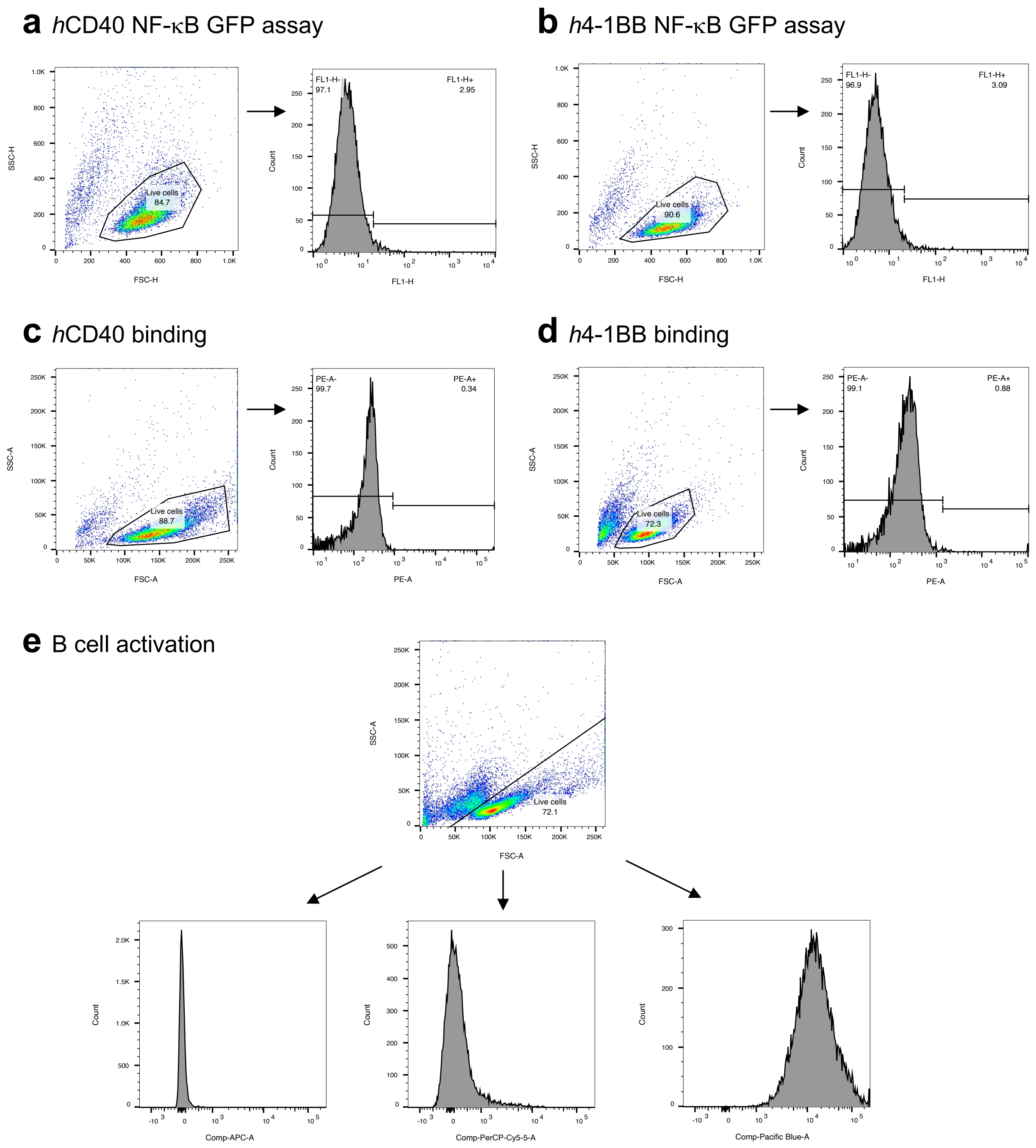

Supplementary Figure 20. Gating strategies for flow cytometry experiments.
Gating strategies shown for the **a** *h*CD40 NF-κB GFP reporter assay, **b** *h*4-1BB NF-κB GFP reporter assay, **c** *h*CD40 cell surface receptor binding assay, **d** *h*4-1BB cell surface receptor binding assay and **e** B cell activation assay.

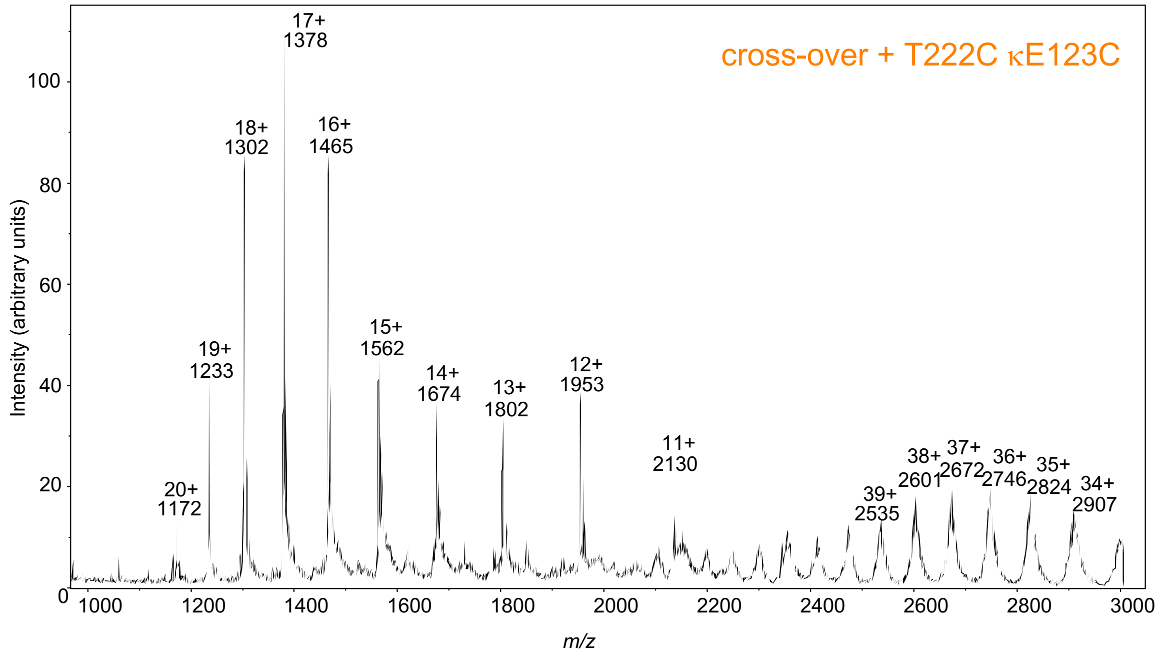

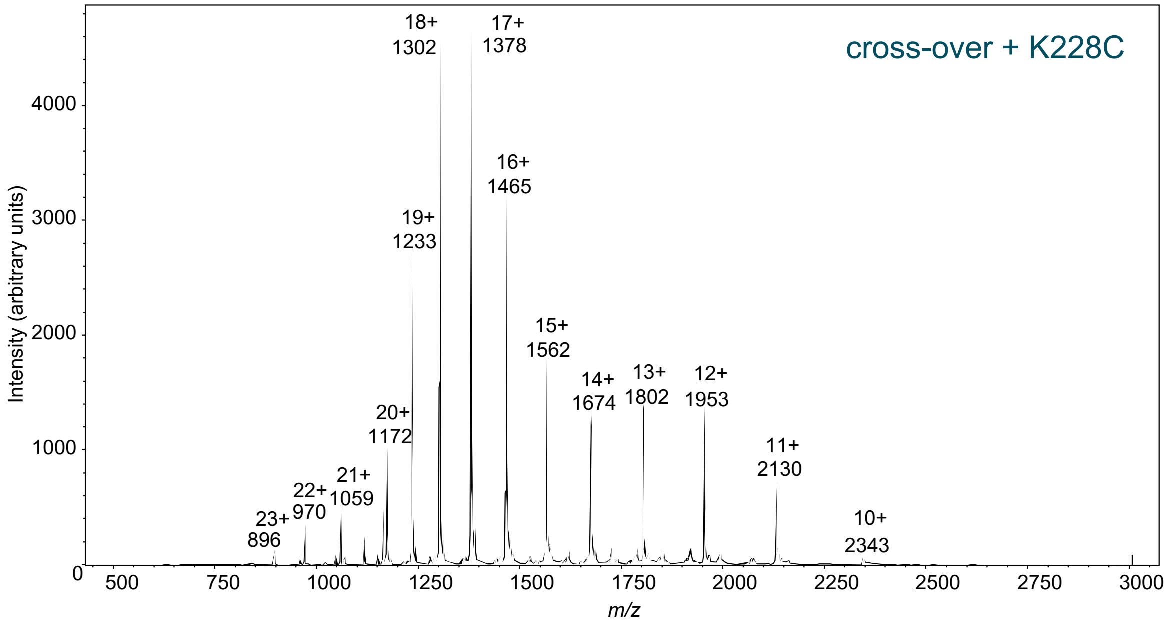

Supplementary Figure 21. Mass spectrometry analysis of anti-*h*CD40 ChiLob7/4 engineered disulfide variant F(ab')$_2$ fragments. F(ab')$_2$ fragments of anti-*h*CD40 disulfide variants were analysed by mass spectrometry. Graphs show positive ion electrospray mass spectra for each F(ab')$_2$. Engineered antibody variants labelled by colour: blue C232S κC214S, teal cross-over + K228C, orange cross-over + T222C κE123C. Source data are provided as a Source Data file.

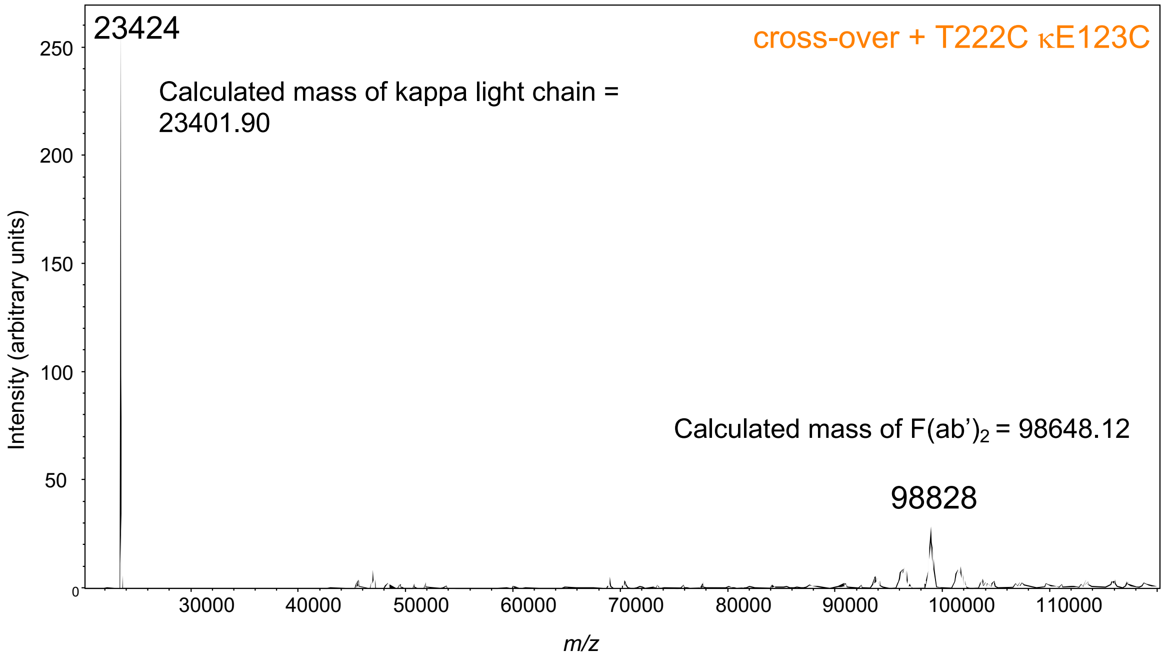

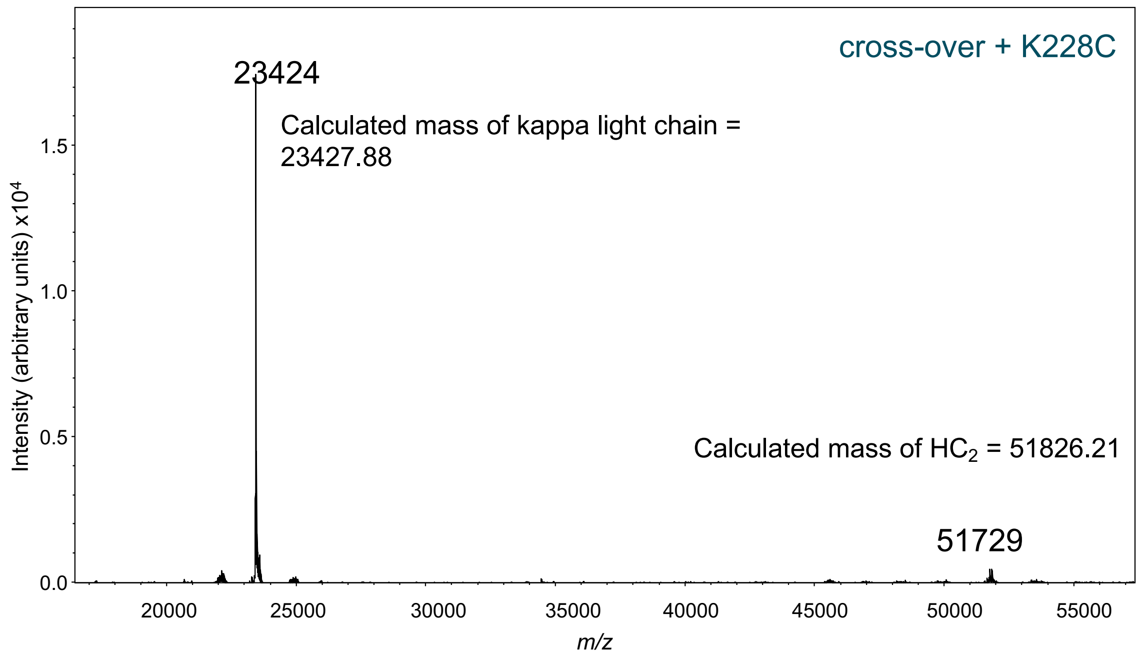

Supplementary Figure 22. Mass spectrometry analysis of anti-*h*CD40 ChiLob7/4 engineered disulfide variant F(ab')$_2$ fragments. F(ab')$_2$ fragments of anti-*h*CD40 disulfide variants were analysed by mass spectrometry. Graphs show MaxEnt deconvoluted spectra identifying the major species for each F(ab')$_2$ along with the experimentally determined masses. This is compared to calculated masses based on amino acid sequence, calculated using Expasy ProtParam[24]. Engineered antibody variants labelled by colour: blue C232S κC214S, teal cross-over + K228C, orange cross-over + T222C κE123C. Source data are provided as a Source Data file.

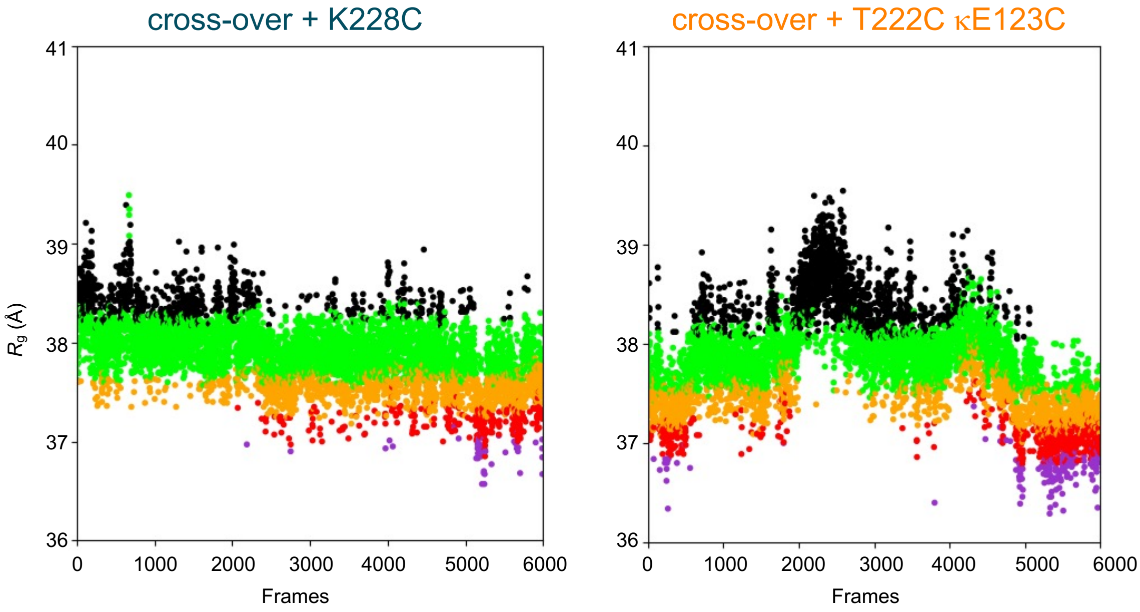

Supplementary Figure 23. Molecular dynamics simulation trajectory analysis. Three independent simulations of 2 μs were performed for cross-over + K228C and cross-over + T222C κE123C as F(ab')$_2$. The three simulations were concatenated and frames were extracted every 1 ns. R_g was calculated using CRYSOL for each of the extracted frames. Frames are coloured by χ^2 agreement to the SAXS data. $\chi^2 < 2$ (black), $2 \leq \chi^2 < 4$ (green), $4 \leq \chi^2 < 6$ (orange), $6 \leq \chi^2 < 8$ (red), $\chi^2 > 8$ (purple). Source data are provided as a Source Data file.

Supplementary References

1 Von Stetten, D. *et al.* ID30A-3 (MASSIF-3) – a beamline for macromolecular crystallography at the ESRF with a small intense beam. *Journal of Synchrotron Radiation* **27**, 844-851, doi:10.1107/s1600577520004002 (2020).

2 Duman, R. *et al.* Sample Preparation and Transfer Protocol for In-Vacuum Long-Wavelength Crystallography on Beamline I23 at Diamond Light Source. *Journal of Visualized Experiments*, doi:10.3791/62364 (2021).

3 Monaco, S. *et al.* Automatic processing of macromolecular crystallography X-ray diffraction data at the ESRF. *Journal of Applied Crystallography* **46**, 804-810, doi:10.1107/s0021889813006195 (2013).

4 Kabsch, W. XDS. *Acta Crystallographica Section D Biological Crystallography* **66**, 125-132, doi:10.1107/s0907444909047337 (2010).

5 Evans, P. R. An introduction to data reduction: space-group determination, scaling and intensity statistics. *Acta Crystallographica Section D Biological Crystallography* **67**, 282-292, doi:10.1107/s090744491003982x (2011).

6 Winter, G. *et al.* DIALS: implementation and evaluation of a new integration package. *Acta Crystallographica Section D Structural Biology* **74**, 85-97, doi:10.1107/s2059798317017235 (2018).

7 Potterton, L. *et al.* CCP4i2: the new graphical user interface to the CCP4 program suite. *Acta Crystallographica Section D Structural Biology* **74**, 68-84, doi:10.1107/s2059798317016035 (2018).

8 Agirre, J. *et al.* The CCP4 suite: integrative software for macromolecular crystallography. *Acta Crystallographica Section D Structural Biology* **79**, 449-461, doi:10.1107/s2059798323003595 (2023).

9 Vagin, A. & Teplyakov, A. Molecular replacement with MOLREP. *Acta Crystallogr D Biol Crystallogr* **66**, 22-25, doi:10.1107/s0907444909042589 (2010).

10 Emsley, P., Lohkamp, B., Scott, W. G. & Cowtan, K. Features and development of Coot. *Acta Crystallographica Section D Biological Crystallography* **66**, 486-501, doi:10.1107/s0907444910007493 (2010).

11 Murshudov, G. N. *et al.* REFMAC5 for the refinement of macromolecular crystal structures. *Acta Crystallographica Section D Biological Crystallography* **67**, 355-367, doi:10.1107/s0907444911001314 (2011).

12 Joosten, R. P., Long, F., Murshudov, G. N. & Perrakis, A. The PDB_REDO server for macromolecular structure model optimization. *IUCrJ* **1**, 213-220, doi:10.1107/s2052252514009324 (2014).

13 Chen, V. B. *et al.* MolProbity: all-atom structure validation for macromolecular crystallography. *Acta Crystallographica Section D Biological Crystallography* **66**, 12-21, doi:10.1107/s0907444909042073 (2010).

14 Burley, S. K. *et al.* Protein Data Bank: the single global archive for 3D macromolecular structure data. *Nucleic Acids Research* **47**, D520-D528, doi:10.1093/nar/gky949 (2019).

15 Afonine, P. V. *et al.* Towards automated crystallographic structure refinement with phenix.refine. *Acta Crystallographica Section D Biological Crystallography* **68**, 352-367, doi:10.1107/s0907444912001308 (2012).

16 Thorn, A. & Sheldrick, G. M. ANODE: anomalous and heavy-atom density calculation. *Journal of Applied Crystallography* **44**, 1285-1287, doi:10.1107/s0021889811041768 (2011).
17 Tria, G., Mertens, H. D. T., Kachala, M. & Svergun, D. I. Advanced ensemble modelling of flexible macromolecules using X-ray solution scattering. *IUCrJ* **2**, 207-217, doi:10.1107/s205225251500202x (2015).
18 Hopkins, J. B. BioXTAS RAW 2: new developments for a free open-source program for small-angle scattering data reduction and analysis. *Journal of Applied Crystallography* **57**, 194-208, doi:10.1107/s1600576723011019 (2024).
19 Manalastas-Cantos, K. *et al.* ATSAS 3.0: expanded functionality and new tools for small-angle scattering data analysis. *Journal of Applied Crystallography* **54**, 343-355, doi:10.1107/s1600576720013412 (2021).
20 Rambo, R. P. & Tainer, J. A. Accurate assessment of mass, models and resolution by small-angle scattering. *Nature* **496**, 477-481, doi:10.1038/nature12070 (2013).
21 Piiadov, V., Ares De Araújo, E., Oliveira Neto, M., Craievich, A. F. & Polikarpov, I. SAXSMoW 2.0: Online calculator of the molecular weight of proteins in dilute solution from experimental SAXS data measured on a relative scale. *Protein Science* **28**, 454-463, doi:10.1002/pro.3528 (2019).
22 Franke, D., Jeffries, C. M. & Svergun, D. I. Machine Learning Methods for X-Ray Scattering Data Analysis from Biomacromolecular Solutions. *Biophysical Journal* **114**, 2485-2492, doi:10.1016/j.bpj.2018.04.018 (2018).
23 Hajizadeh, N. R., Franke, D., Jeffries, C. M. & Svergun, D. I. Consensus Bayesian assessment of protein molecular mass from solution X-ray scattering data. *Scientific Reports* **8**, doi:10.1038/s41598-018-25355-2 (2018).
24 E., G. *et al.* in *The Proteomics Protocols Handbook* (ed John M. Walker) Ch. Protein Identification and Analysis Tools on the Expasy Server, 571-607 (Humana Press, 2005).
25 Tully, M. D. *et al.* BioSAXS at European Synchrotron Radiation Facility – Extremely Brilliant Source: BM29 with an upgraded source, detector, robot, sample environment, data collection and analysis software. *Journal of Synchrotron Radiation* **30**, 258-266, doi:10.1107/s1600577522011286 (2023).
