# Supplementary material for: Structure-guided disulfide engineering restricts antibody conformation to elicit TNFR agonism
Source: Nat Commun. 2025 Apr 12;16:3495. doi: 10.1038/s41467-025-58773-8 (PMC11993666; doi:10.1038/s41467-025-58773-8)
Supplement: Supplementary file 2 — Reporting Summary [file 41467_2025_58773_MOESM2_ESM.pdf]

nature portfolio

Corresponding author(s): Ivo Tews

Last updated by author(s): Feb 27, 2025

Reporting Summary

Nature Portfolio wishes to improve the reproducibility of the work that we publish. This form provides structure for consistency and transparency in reporting. For further information on Nature Portfolio policies, see our Editorial Policies and the Editorial Policy Checklist.

Statistics

For all statistical analyses, confirm that the following items are present in the figure legend, table legend, main text, or Methods section.

n/a	Confirmed	
☐	☒	The exact sample size (*n*) for each experimental group/condition, given as a discrete number and unit of measurement
☐	☒	A statement on whether measurements were taken from distinct samples or whether the same sample was measured repeatedly
☐	☒	The statistical test(s) used AND whether they are one- or two-sided<br>*Only common tests should be described solely by name; describe more complex techniques in the Methods section.*
☐	☒	A description of all covariates tested
☐	☒	A description of any assumptions or corrections, such as tests of normality and adjustment for multiple comparisons
☐	☒	A full description of the statistical parameters including central tendency (e.g. means) or other basic estimates (e.g. regression coefficient) AND variation (e.g. standard deviation) or associated estimates of uncertainty (e.g. confidence intervals)
☐	☒	For null hypothesis testing, the test statistic (e.g. *F*, *t*, *r*) with confidence intervals, effect sizes, degrees of freedom and *P* value noted<br>*Give P values as exact values whenever suitable.*
☒	☐	For Bayesian analysis, information on the choice of priors and Markov chain Monte Carlo settings
☒	☐	For hierarchical and complex designs, identification of the appropriate level for tests and full reporting of outcomes
☒	☐	Estimates of effect sizes (e.g. Cohen's *d*, Pearson's *r*), indicating how they were calculated

Our web collection on statistics for biologists contains articles on many of the points above.

Software and code

Policy information about availability of computer code

Data collection	BD CellQuest Pro (Version 6.1) and BD FACSDIVA (Version 9) was used to collect flow cytometry data<br>Biacore T200 control software (Version 2.0.2) was used to collect SPR data<br>MXCuBE3 (https://mxcube3.esrf.fr/) was used for acquisition of X-ray crystallography data<br>BsxCuBE3 was used for acquisition of SAXS data
Data analysis	Biacore T200 Evaluation software (Version 3.1) was used to analyse SPR data<br>ThermControl software (NanoTemper Technologies) first derivative analysis of nano differential scanning fluorimetry (NanoDSF) data<br>GraphPad Prism (Version 10.0.3) was used for data analysis and to perform statistical tests<br>FlowJo (Version 10.8.1) was used to analyse flow cytometry data<br>GrenADES ParallelProc (Version Jan 10, 2022 BUILT=20220110), and XIA2 DIALS (XIA2 Version 3.10.devO, DIALS Version 3.dev, CCP4 Version 7.1.018) were used for processing of crystallography data<br>CCP4i2 (Version 8) was used for X-ray crystal structure determination (MOLREP for molecular replacement, Coot for model building, and Refmac for refinement, MolProbity for model validation)<br>PDB-Redo server (https://pdb-redo.eu/) was used to generate restraints for refinement<br>PDB Validation Server (https://validate-rcsb-2.wwpdb.org/) were used for model validation<br>Pymol (Version 2.5.0) was used to analyse X-ray crystallography data and visualise molecular dynamics simulations<br>ATSAS (Version 3.1.1, including CRYSOL, PRIMUS) was used to analyse SAXS data<br>BioXTAS RAW 2.3.019 for Guinier analysis of SAXS data<br>GNOM from ATSAS 4.0.1-120 implemented in BioXTAS RAW 2.3.0 for P(r) analysis of SAXS data

ProtParam (https://web.expasy.org/protparam/) was used to calculate molecular weight from chemical composition
GAJOE (Version 2.1) was used to analyse molecular dynamics (MD) simulations
MODELLER (Version 10.2) was used for crystal structure model completion prior to MD simulations
H++ server (http://newbiophysics.cs.vt.edu/H++/) and PDB2PQR (https://server.poissonboltzmann.org/pdb2pqr) were used to assign protonation states prior to MD simulations
GROMACS (Version 2022.4) were used to perform MD simulations
Bruker OtofControl Acquisition Program version 3.2.41.0 and Instrument Program version 5.3.14.0 were used to collect mass spec data
Bruker DataAnalysis version 4.0.275.0 was used to analyse mass spec data

For manuscripts utilizing custom algorithms or software that are central to the research but not yet described in published literature, software must be made available to editors and reviewers. We strongly encourage code deposition in a community repository (e.g. GitHub). See the Nature Portfolio guidelines for submitting code & software for further information.

Data

Policy information about availability of data

All manuscripts must include a data availability statement. This statement should provide the following information, where applicable:

- Accession codes, unique identifiers, or web links for publicly available datasets
- A description of any restrictions on data availability
- For clinical datasets or third party data, please ensure that the statement adheres to our policy

Crystallographic data has been deposited in the Protein Data Bank (PDB) with accession codes 8PUL and 8PUK (Supp Table 2). SAXS data has been deposited in the Small Angle Scattering Biological Data Bank (SASBDB) with accession codes SASDUB8, SASDUC8, SASDUD8, SASDUE8, SASDUF8, SASDUG8, SASDUH8, SASDUJ8, SASDUK8, SASDUL8, SASDSC7, SASDSD7, SASDWN2, SASDWP2, SASDWQ2, SASDWR2, SASDWS2, SASDWT2, SASDWU2, SASDWV2, SASDWW2, SASDWX2, SASDWY2 and SASDWZ2. MD simulation data is available at doi.org/10.5281/zenodo.12582681. Raw SAXS data for BM29, proposal MX2373 is available at doi.org/10.15151/ESRF-ES-748850843 and doi.org/10.15151/ESRF-ES-771372332 and for BM29, MX2639 is available at doi.org/10.15151/ESRF-ES-1830158910 and doi.esrf.fr/10.15151/ESRF-ES-1893933559. Raw crystallography X-ray data is available at doi.org/10.15151/ESRF-ES-686689060 for ID30A-3, proposal MX2373, at https://ispyb.diamond.ac.uk/dc/visit/mx29835-1/id/8064344 for I23, proposal number MX29835-1 and at https://ispyb.diamond.ac.uk/dc/visit/mx29835-9/id/8379953 for I23, proposal number MX29835-9. All other data needed to evaluate the conclusions in the paper are present in the paper or the Supplementary Materials. Request for materials will be subject to a standard MTA with the University of Southampton.

Research involving human participants, their data, or biological material

Policy information about studies with human participants or human data. See also policy information about sex, gender (identity/presentation), and sexual orientation and race, ethnicity and racism.

Reporting on sex and gender	No human research participants were engaged directly during the study. Human blood cones were obtained from anonymous healthy donors providing platelet donations to the NHS blood transfusion service. Samples were derived from both males and females. Due to the size of the donation required, ~90% of the donors are males
Reporting on race, ethnicity, or other socially relevant groupings	n/a
Population characteristics	No human research participants were engaged directly during the study. Human blood cones were obtained from anonymous healthy donors providing platelet donations to the NHS blood transfusion service. Samples were derived from both males and females. Due to the size of the donation required, ~90% of the donors are males
Recruitment	Anonymous donors provide blood to the NHS blood transfusion service with blood products provided for use to ethically approved studies with prior consent.
Ethics oversight	The use of human blood was approved by the East of Scotland Research Ethics Service, Tayside, UK.

Note that full information on the approval of the study protocol must also be provided in the manuscript.

Field-specific reporting

Please select the one below that is the best fit for your research. If you are not sure, read the appropriate sections before making your selection.

☒ Life sciences ☐ Behavioural & social sciences ☐ Ecological, evolutionary & environmental sciences

For a reference copy of the document with all sections, see nature.com/documents/nr-reporting-summary-flat.pdf

Life sciences study design

All studies must disclose on these points even when the disclosure is negative.

Sample size	No statistical methods were used to predetermine sample size in in vitro experiments. For in vitro assays (to assess cell binding and immunostimulatory activity using cell lines), experiments were performed at least two times, with each independent experiment consisting of sample triplicates to control technical variations. For experiments using human PBMCs, experiments were repeated with three different donors (with each independent experiment involving triplicate wells). Human PBMC data is plotted for 1 donor only (data representative of all 3 donors) due to expected biological variation between donors (we saw the same trends between all three donors, but actual values varied).

	For X-ray crystallography experiments, sample size is not applicable as novel crystal structures are reported only. For molecular dynamics simulations, three independent equilibration runs were performed with different random number seeds for velocity generation, from which three independent molecular dynamics production runs were performed. For SAXS experiments, SEC-SAXS was performed to ensure sample was monodisperse, and allowing SAXS measurements to be taken from a well-resolved chromatographic peak of a single protein species.
Data exclusions	No data were excluded from the analysis.
Replication	For in vitro experiments, typically three and at least two independent experiments were performed. For molecular dynamics simulations, three independent production runs were performed. Details of experimental replicates are stated in the figure legends. All attempts at replication were successful.
Randomization	Antibody treatments to be compared were added to each cell sample or cell-line and assessed within each experiment, allowing clear conclusions to be made. As such there were no defined experimental groups per se.
Blinding	A single investigator typically performed each experiment, not blinded to experimental groups. We do not have sufficient resource to have a second person available to assess samples (e.g. through an independent flow cytometrist through a flow cytometry core service) in a blinded manner routinely.

Reporting for specific materials, systems and methods

We require information from authors about some types of materials, experimental systems and methods used in many studies. Here, indicate whether each material, system or method listed is relevant to your study. If you are not sure if a list item applies to your research, read the appropriate section before selecting a response.

Materials & experimental systems

n/a	Involved in the study
☐	☒ Antibodies
☐	☒ Eukaryotic cell lines
☒	☐ Palaeontology and archaeology
☒	☐ Animals and other organisms
☒	☐ Clinical data
☒	☐ Dual use research of concern
☒	☐ Plants

Methods

n/a	Involved in the study
☒	☐ ChIP-seq
☐	☒ Flow cytometry
☒	☐ MRI-based neuroimaging

Antibodies

Antibodies used	The following antibodies were produced in house:<br>Anti-CD40 ChiLob7/4 hIgG1, anti-CD40 ChiLob7/4 hIgG2, anti-CD40 ChiLob7/4 hIgG2 C232S+C233S, anti-CD40 hIgG2 C232S kC214S, anti-CD40 hIgG2 C233S kC214S, anti-CD40 hIgG2 C232S+K228C kC214S, anti-CD40 hIgG2 C232S+T222C kE123C+kC214S, anti-4-1BB SAP1.3 hIgG1, anti-4-1BB SAP1.3 hIgG2, anti-4-1BB SAP1.3 hIgG2 C232S+C233S, anti-4-1BB SAP1.3 hIgG2 C232S kC214S, anti-4-1BB SAP1.3 C233S kC214S.<br>The following antibodies were purchased from commercial suppliers: anti-hCD23 APC, BioLegend, clone EBVCS-5, Cat#338514<br>anti-hCD86 PerCP/Cyanine5.5, BioLegend, clone BU63, Cat#374216<br>anti-hHLA-DR Brilliant Violet 421, BioLegend, clone L243, Cat#307636<br>R-Phycoerythrin-conjugated AffiniPure F(ab')2 Fragment Goat Anti-Human IgG, Fcγ Fragment Specific (min X Bov, Hrs, Ms Sr Prot), Jackson ImmunoResearch Europe Ltd., Cat#109-116-098
Validation	The specificity of commercial antibodies was verified by the manufacturer, in-house antibodies were verified using cell lines specific to their species specificity and data in this manuscript support their specificity. All in-house antibodies are subjected to rigorous QC including being checked by HPLC to contain < 1% aggregate and by Endosafe-PTS portable test (Charles River Laboratories) to contain < 5EU endotoxin/mg antibody.

Eukaryotic cell lines

Policy information about cell lines and Sex and Gender in Research

Cell line source(s)	Jurkat-NFkB-GFP reporter (System Biosciences)
Authentication	Cell lines were purchased from commercial suppliers with clear provenance so no further authentication was performed.
Mycoplasma contamination	Mycoplasma test were conducted using the Mycoplasma: MycoAlert Mycoplasma Detection Kit (Lonza) and returned negative results.
Commonly misidentified lines (See ICLAC register)	None used in this study.

Plants

Seed stocks	*Report on the source of all seed stocks or other plant material used. If applicable, state the seed stock centre and catalogue number. If plant specimens were collected from the field, describe the collection location, date and sampling procedures.*
Novel plant genotypes	*Describe the methods by which all novel plant genotypes were produced. This includes those generated by transgenic approaches, gene editing, chemical/radiation-based mutagenesis and hybridization. For transgenic lines, describe the transformation method, the number of independent lines analyzed and the generation upon which experiments were performed. For gene-edited lines, describe the editor used, the endogenous sequence targeted for editing, the targeting guide RNA sequence (if applicable) and how the editor was applied.*
Authentication	*Describe any authentication procedures for each seed stock used or novel genotype generated. Describe any experiments used to assess the effect of a mutation and, where applicable, how potential secondary effects (e.g. second site T-DNA insertions, mosiacism, off-target gene editing) were examined.*

Flow Cytometry

Plots

Confirm that:

☐ The axis labels state the marker and fluorochrome used (e.g. CD4-FITC).

☒ The axis scales are clearly visible. Include numbers along axes only for bottom left plot of group (a 'group' is an analysis of identical markers).

☐ All plots are contour plots with outliers or pseudocolor plots.

☐ A numerical value for number of cells or percentage (with statistics) is provided.

Methodology

Sample preparation	Cells were prepared for flow cytometry by centrifugation at 650 g for 2 minutes or 450 g for 5 mins and resuspended in PBS or PBS with 1% BSA, 0.01% sodium azide
Instrument	Flow cytometry was performed using either a FACS Calibur or FACS Canto II (BD Biosciences).
Software	FACS Calibur data were collected using BD Cell Quest and FACS Canto II data were collected using BD FACSDIVA. Data were analysed using FlowJo.
Cell population abundance	Cell sorting was not performed. For experiments, 10,000 cells were collected, and samples were gated based on their FSC/SSC properties.
Gating strategy	FSC/SCC gates were based on prior knowledge of the position of viable cells in a population based on these parameters due to the cell size and gran.ularity. Positive cell populations were selected based on the observation of distinct populations that were stained for the cell marker being probed versus staining with an isotype control. Typical flow histograms of fluorescent antibody staining and GFP responses are shown in the main and supplementary figures.

☒ Tick this box to confirm that a figure exemplifying the gating strategy is provided in the Supplementary Information.
